# Supplementary material for: Impact of digital meditation on work stress and health outcomes among adults with overweight: A randomized controlled trial
Source: PLoS One. 2023 Mar 1;18(3):e0280808. doi: 10.1371/journal.pone.0280808 (PMC9977041; doi:10.1371/journal.pone.0280808)
Supplement: S1 File — (DOCX) [file pone.0280808.s002.docx]

**Stress Free UC Study & Stress Free UC +Health Study (SFUC Study and +Health Substudy)**

**Clinicaltrials.gov #** **NCT03945214**

**Study Protocol**

**(list the headers, and subheaders, fill in what you can)**

| **Table 1. The CONSORT 2010 and -SPI 2018 checklist with SFUC Content** | | | | |
| --- | --- | --- | --- | --- |
| **Section** | **Item #** | **CONSORT 2010** | **CONSORT-SPI 2018** | **SFUC Content** |
| Title and abstract | 1a | Identification as a randomised trial in the title |  | Title: Stress Free UC Study |
|  | 1b | Structured summary of trial design, methods, results, and conclusions (for specific guidance, see CONSORT for Abstracts) | Refer to CONSORT extension for social and psychological intervention trial abstracts | See abstract. |
| Introduction |  |  |  |  |
| Background and objectives | 2a | Scientific background and explanation of rationale |  | High levels of psychosocial work-related stress have major implications for both the employee and the employer.  Epidemiological studies consistently demonstrate associations between high work stress and worse self-reported mental and physical health, including depression, anxiety, cardiovascular disease, and type 2 diabetes (Ganster & Rosen, 2013).  In the United States, it’s estimated that 5-8% of annual healthcare costs are attributable to work-related stressors (Goh, Pfeffer, & Zenios, 2016).  Job strain, a combination of high demands (workload and intensity) and low control (discretion over work tasks), is one of the most widely studied models used to define psychosocial stress at work (Karasek, 1979).  Epidemiological studies and meta-analyses of decades of research have found that job strain is associated with worse mental and physical health, including anxiety and depressive disorders, increased blood pressure, cardiovascular events, and metabolic syndrome (Chandola, Brunner, & Marmot, 2006; Landsbergis, Dobson, Koutsouras, & Schnall, 2013; Madsen et al., 2017; Steptoe & Kivimäki, 2013).              The potential stress-reduction and psychological well-being benefits of teaching mindfulness in the workplace has received increased attention and initial empirical support (Jamieson & Tuckey, 2017). Meta-analyses have reported that mindfulness-based psychological interventions decrease stress in healthy non-clinical populations, and improve psychosocial outcomes for people with clinical disorders such as anxiety and depression (Bohlmeijer, Prenger, Taal, & Cuijpers, 2010; Chiesa & Serretti, 2009; Hofmann, Heering, Sawyer, & Asnaani, 2009; Kuyken, Warren, Taylor, & Whalley, 2016).              Mindfulness training delivered via self-guided smartphone app may offer a convenient alternative to group sessions.  App-based treatments to improve mental health are an increasingly popular method of service delivery, though research on the efficacy of these apps is limited (Donker et al., 2013; Fairburn & Patel, 2017).  Initial evidence of technology-driven delivery of standard treatment protocols for clinical disorders such as Cognitive Behavioral Therapy for anxiety have demonstrated effect sizes comparable to conventional standard of care (e.g. Berger, Boettcher, & Caspar, 2014; Titov et al., 2011).  Three small studies using smartphone apps to deliver mindfulness interventions to healthy adults found benefits comparable to traditional delivery methods on outcomes of subjective well-being, depressive symptoms, and compassion (Howells, Ivtzan, & Eiroa-Orosa, 2016; Lim, Condon, & De Steno, 2015; Ly et al., 2014). |
|  | 2b | Specific objectives or hypotheses. | If pre-specified, how the intervention was hypothesised to work | The aim of the present study is to test the effects of a digital meditation intervention in a sample of high stress UCSF employees. We propose to randomize 1000 UCSF employees to 8-weeks of either a digital mindfulness intervention (using the commercially available application Headspace) or a waitlist control condition. Measurement time points include baseline, week 4, week 8 (post intervention), and 4-month follow up period.  Among participants who are randomized to the digital meditation intervention, they will also take part in a 1-year follow up. Our primary outcomes will be reductions in global levels of perceived stress and job strain. Secondary outcomes include improvements in mindfulness, symptoms of anxiety and depression, health behaviors, including sleep, physical activity, and eating behavior, and health care utilization.  Within this larger study, we will also conduct a smaller more focused substudy in which we will recruit overweight and obese participants (BMI => 25). This study will include all of the measures obtained in the larger, main study but also expand to include physiological assessments, personal fitness and sleep metrics, and measures of daily mood data. The physiological assessments will take place at baseline and week 8, and the fitness, sleep, and mood data will be gathered for seven consecutive days at baseline and week 7. All other measurement time points are similar to those of the main study. For this substudy, we will recruit 150 additional UCSF employees, who will be randomly assigned to one of four conditions: digital meditation, a healthy eating intervention, digital meditation+healthy eating intervention, or a waitlist control condition. If participants are assigned to either of the Healthy Eating conditions, in addition to the above-mentioned assessments, they will participate in one 30- to 60-minute counseling session centered around healthy eating behaviors such as decreasing sugar intake or the frequency of stress-related eating, and three 10-minute booster phone calls at week 1, week 4, and week 8. |
| Methods |  |  |  |  |
| Trial Design | 3a | Description of trial design (such as parallel, factorial) including allocation ratio^§^ | If the unit of random assignment is not the individual, please refer to CONSORT for Cluster Randomised Trials [[8](https://www.ncbi.nlm.nih.gov/pmc/articles/PMC6066913/#CR8)] | We propose a randomized controlled trial testing the benefits of an 8-week digital intervention compared to a waitlist control condition in 2,000 high stress (i.e., perceived stress score of 15 or higher) UCSF employees. Eligible participants will complete a baseline assessment and then undergo randomization. Participants randomized to the digital meditation condition will use the smartphone/computer application called Headspace which provides digital audio mediations that are 10 minutes in duration.  There will be a midpoint assessment at week 4 of the intervention, a post-intervention assessment at week 8, and a follow-up assessment +4 months from baseline.  Those randomized to the waitlist control condition will then be provided access to the digital meditation condition. All questionnaires will be provided electronically and there will be no in person assessments. Among participants initially randomized to the digital meditation condition there will be an additional 1-year follow-up questionnaire to assess durability of the intervention.    We propose an additional sample of 150 participants to examine a mindfulness program along with a healthy eating program. Eligible participants (with a BMI of 25 or above) who have consented to this sub-study, will complete online surveys, as well as in-person clinic assessments. At these assessments, study staff will orient participants to the study, collect anthropometric (e.g., weight, waist to hip ratio) and physiological measurements (e.g., fasting glucose) at pre-and post-intervention. Then, subjects will complete the baseline questionnaire online. One week prior to randomization, participants will complete twice-daily surveys in the morning and at night for 7 days, while wearing a fitness watch that collects various measures such as physical activity and sleep. Participants randomized to the digital meditation condition will use the smartphone/computer application called Headspace which provides digital audio mediations that are 10 minutes in duration. Those randomized to the waitlist control condition will then be provided access to the digital meditation condition. Participants may also take part in a healthy eating program designed to assess and reduce problematic eating behavior such as stress-related eating. There will be a midpoint assessment at week 4 of the intervention, a post-intervention assessment at week 8, and a follow-up assessment 4 months from baseline. During week 7 of the intervention, participants will once again be asked to complete the weeklong daily and nightly diaries and wear the fitness watch. At week 8, they will partake in a second in-person clinical visit. Among participants initially randomized to the digital meditation condition there will be an additional 1-year follow-up questionnaire to assess durability of the intervention. |
|  | 3b | Important changes to methods after trial commencement (such as eligibility criteria), with reasons |  | The sub study was introduced as a supplemental component of the larger Stress Free UC Study. The modification was approved in October of 2018. |
| Participants | 4a | Eligibility criteria for participants^§^ | When applicable, eligibility criteria for settings and those delivering the interventions | Participants were drawn from of UCSF employees (faculty and staff). Participants were targeted at UCSF campuses and utilizing university services such as flyers, advertisements, digital media, emails through listservs.  Those interested in participation can inquire on the website for more details, and their eligibility for the study will be determined by the screening questionnaire. If they are eligible, they will have the option to consent and will be invited into the study via an automated email.  . Inclusion criteria were:   - Have access to a smartphone or computer every day - Fluent in English and has at least a third grade reading level - Is a UCSF employee - Perceived Stress Scale total of score of 15 or higher - Consent: demonstrates understanding of the study and willingness to participate as evidenced by voluntary informed consent and has received a signed and dated copy of the informed consent form - Is at least 18 years of age - Expresses willingness to be randomly assigned into with the waitlist group or the intervention group   Substudy participants must report a BMI of 25 or above.  Exclusion criteria were:   - Experienced meditator and/or report current (three times per week or more- for 10 minutes at a time) sitting meditiation practice   There were no inclusion or exclusion criteria based on gender, race or ethnicity. |
|  | 4b | Settings and locations where the data were collected |  | All activities- screening, registration, surveys, etc., will take place online. Participants can access questionnaires, assessments, and all other study materials on their own time on a computer or smartphone.    Sub-study: Screening, surveys, and digital components of the intervention will take place online. The in-person clinic visits at baseline and week 8 will take place at the Parnassus campus at Langley Porter Psychiatric Institute. Counseling sessions will be scheduled in a private room at the UCSF campus at which the participant works. |
| Interventions | 5 | The interventions for each group with sufficient details to allow replication, including how and when they were actually administered^§^ |  | The main study is designed to be carried out over the internet and to be as automated as possible. Interactions with participants will be facilitated online, by email, and, if necessary, by phone. As will be described below, the sub-study will require in person assessments.  Screening: Individuals interested in learning more and potentially participating will log on to a secure website. Information about the study will be presented.  If the individual is interested in joining the study he/she will complete questions relevant to our inclusion and exclusion criteria as well as sociodemographic questions (age, sex, and self-reported height and weight).  Main study: Based on the eligibility criteria for the larger study, participants will be directed to the study consent document, and if voluntary consent is given, they will be provided a link to the baseline battery. Participants agreeing to release their medical records will be directed to the PHI form on Docusign, where they will be asked to review and then sign and date the document.  Sub-study: If a participant meets the eligibility requirements for the sub-study, he or she will receive a message offering him/her the opportunity to participate. After reviewing the procedures and learning more about the procedures, a participant will have the option to join this study. If they decline participation, they will still have the opportunity to participate in the main study. If interested in the sub-study, they will be routed into a group for sub-study participants and will expect to hear from study staff to schedule an in person visit.  Clinic-visit (sub-study only): Participants interested in joining the sub-study will be contacted by a study staff member, who will guide them through the procedures and answer any questions that arise. Participants will then be scheduled for their first in-person clinic visit. The staff member will review the details of the visit, such as how to prepare and where to go.  The clinic visit assessment will consist of the following activities:   - Read and sign consent form - Height/Weight measurement - to calculate BMI (confirm eligibility) - Blood Pressure collection - Blood Spot collection - Waist to Hip Ratio collection - Instruction on Morning and Nightly Diary procedures - Orientation on Fitbit procedures   Baseline Questionnaire Battery (main study). This battery will be hosted by Qualtrics and all data will be secure. As part of this battery, participants will complete questionnaires to assess stress/distress symptoms as well the domain areas of mindfulness, health behaviors, physical health and work impact. Once completed, participants will be randomized to one of two groups: digital meditation group (Headspace) or waitlist control.  Baseline Questionnaire (Sub-study): The baseline questionnaire will be sent out to participants shortly after their visit, to complete on their own. Domain areas will be similar to the main study, with the addition of several health and eating behavior components.  Fitbit device (sub-study only): During the week prior to randomization and week 7 of the intervention period, participants will be asked to wear a Fitbit watch 24-hours per day. The Fitbit watch tracks information such as physical activity, sleep, heart rate and movement. Participants are asked to wear it at all times possible, with the exception of charging, cleaning, and syncing their device.  Daily Diary (sub-study only): During the week prior to randomization and week 7 of the intervention period, participants are asked to complete a nightly diary and morning diary for 7 consecutive days. These diaries ask questions related to daily behaviors, sleep, mood and other related topics (see attached file).  Randomization (main-study)  Upon completing the baseline questionnaire, participants enrolled in the main study will be randomized to the following conditions:  Digital Meditation Condition (Headspace): The intervention period will last for a total of 8-weeks (56 ± 2 days). Subjects in the Headspace group will be given simple instruction on how they should begin the journey (i.e. begin with Basics 1 followed by the Stress Pack, one 10 minute session per day). Subjects in the waitlist control group will not be given access to Headspace and will be instructed to refrain from any mindfulness or meditation. Active daily usage data in the Headspace group will be captured anonymously via the Headspace app and provided to UCSF as a part of the final analysis. App usage (engagement) will be tracked weekly for each participant.  Waitlist Condition: Participants in this condition will not receive any further study contact during the study window, with the exception of email links to questionnaire batteries at week 4 (midpoint assessment), week 8 (post-intervention), and 4-month follow-up.  Once the 4-month follow up is completed or that time has lapsed, the study participant will be provided access to Headspace for the same duration (12 months) as is the case for those randomized to the digital meditation group.    Randomization (sub-study):    Upon completing the baseline questionnaire battery and 7 days of morning and nightly diaries and Fitbit measurement, participants will be randomized to one of the following study conditions:    Digital Meditation Condition (Headspace): The intervention period will last for a total of 8-weeks (56 ± 2 days). Subjects in the Headspace group will be given simple instruction on how they should begin the journey (i.e. begin with Basics 1 followed by the Stress Pack, one 10 minute session per day). Subjects in the waitlist control group will not be given access to Headspace and will be instructed to refrain from any mindfulness or meditation. Active daily usage data in the Headspace group will be captured anonymously via the Headspace app and provided to UCSF as a part of the final analysis. App usage (engagement) will be tracked weekly for each participant.    Healthy Eating condition: Those assigned to this condition will participate in a healthy eating program, designed to identify and assist problem eating behaviors, such as stress-related eating, which may contribute to metabolic problems. The program will include a 30- to 60-minute in-person counseling session at the beginning of the 8-week period along with 3 follow up booster phone calls and weekly text messages throughout the program. Following the counseling session, subjects will be given access to 2 online audio exercises to use when experiencing cravings and/or stress prior to eating/meal time. They will be required to access the audio tools at least once per week. One audio exercise uses mindful eating techniques to help participants make healthy eating choices, and the other audio exercise helps participants manage cravings for sweets and other tempting foods. Once the 4-month follow up is completed or that time has lapsed, the study participant will be provided access to Headspace for the same duration (12 months) as is the case for those randomized to the digital meditation group.                Counseling sessions: Once a participant is randomized into a condition that includes a healthy eating program, they will be contacted by phone, informed of their group randomization, offered a brief overview of the program, and coordinate a time and date to meet for an in-person 30- to 60-minute counseling session. In person counseling sessions will be conducted by a trained counselor (see script attached). During the counseling session, topics that will be covered include: a) psychoeducation regarding aspects of eating behaviors that contribute to metabolic dysfunction (e.g., cravings for sweets, stress-related eating, mindless eating), (b) identification of subject’s triggers for overeating, cravings, and non-homeostatic eating behaviors (d) goal-setting to reduce problematic eating behavior, and (c) introduction to mindful eating and cravings management exercises.    Healthy Eating and Digital Meditation Condition: This condition will last for a total of 8-weeks (56 ± 2 days). Subjects in this group will be asked to download and use the Headspace app for 10 minutes per day. Subjects in the Headspace group will be given simple instruction on how they should begin the journey (i.e. begin with Basics 1 followed by the Stress Pack, one 10 minute session per day). Active daily usage data in the Headspace group will be captured anonymously via the Headspace app and provided to UCSF as a part of the final analysis. App usage (engagement) will be tracked weekly for each participant.  Additionally, participants will participate in a healthy eating program, designed to identify and assist problem eating behaviors, such as stress-related eating, which may contribute to metabolic problems. The program will include a 30- to 60-minute in-person counseling session (for more information, see below; these sessions will be audio recorded for treatment fidelity and research purposes) at the beginning of the 8-week period along with 3 follow up booster phone calls and weekly messages throughout the program. Following the counseling session, subjects will be given access to 2 online audio exercises to use when experiencing cravings and/or stress prior to eating/meal time. They will be required to access the audio tools at least once per week. One audio exercise uses mindful eating techniques to help participants make healthy eating choices, and the other audio exercise helps participants manage cravings for sweets.    Waitlist Condition: Participants in this condition will not receive any further study contact during the study window, with the exception of email links to questionnaire batteries at week 4 (midpoint assessment), week 8 (post-intervention), and 4-month follow-up.  Once the 4-month follow up is completed or that time has lapsed, the study participant will be provided access to Headspace for the same duration (12 months) as is the case for those randomized to the digital meditation group.    Midpoint Questionnaire Battery (week 4). This battery will be hosted by Qualtrics and all data will be secure. An email link to this battery will be sent at week 4 for all study participants.  This battery will be an abbreviated version of questionnaires completed at baseline, including stress/distress symptoms as well the domain areas of mindfulness, work impact, and for the sub-study, will include the domain of eating behaviors.    Post-intervention Questionnaire Battery (week 8). This battery will be hosted by Qualtrics and all data will be secure. This battery will serve as the post-intervention assessment and will be comprised of the same questionnaires included at the baseline assessment. Additionally, participants will be asked how often they have participated in a sitting meditation in the past 8 weeks. This battery will be made available to all participants via an email link.    Follow-up Questionnaire Battery (4-month follow-up (i.e., 2-months from end of intervention)).  Participants will receive a brief questionnaire battery similar to the midpoint assessment including measures of stress/distress symptoms as well the domain areas of mindfulness and work impact and health care utilization, and for the sub-study, will include the domain of eating behaviors.    Follow-up Questionnaire Battery (1-year follow-up (i.e., 10-months from end of intervention)). Participants who were initially randomized to the digital meditation condition will complete a brief questionnaire similar to the 4-month follow-up to assess the durability of the intervention, assess domains of distress, mindfulness, and work impact as well as health care utilization. The sub-study participants will also answer questions related to eating behaviors, similar to the baseline assessment.    Medical and employment record data: We are interested in whether meditation practice may affect health care utilization, job performance, and attendance. Participants will be option of making their electronic medical record and employee data available for use in this research study. All data will be collected and stored in a secured fashion, and like all the data in this study, will be deidentified. Participants can decline this request of access and continue to participate in this study.    Re-engagement protocol: As with any digital intervention study, attrition is common. In an effort to minimize attrition in this study, we have developed a re-engagement protocol. We will send an engagement text message to each participant starting about one week into their participation  and for each subsequent week, for 7 weeks. The text message content for the intervention group will consist of gentle reminders to continue to meditate, along with tips to stay active and engaged in the study. For the waitlist control group, these weekly messages will consist of encouragements and brief reminders of participation requirements (see attached doc for specific text). If a participant does not engage after about 2 weeks, then he/she will receive a phone call from our research staff. For those participants who do not answer the phone, the study staff will leave a voicemail and a subsequent text message asking to call them back, and follow up again the next day with another text message. They only hear back from the staff in 2 weeks if they continue to refrain from the meditation app.    Sub-study: Participants will follow the same re-engagement protocol listed above, with minor modifications for the healthy eating groups.    Those in the healthy eating groups will participate in 3 separate booster phone calls (5-10 minutes) to check-in regarding progress and barriers towards healthy eating goals, and feasibility/acceptability of the brief eating intervention. Participants will receive automated text message pings approximately 3 times per week. The pings will provide reminders about mindful eating (including a link to audio sessions on cravings and mindful eating). The pings will occur during identified times of “high vulnerability” from the initial counseling session. |
|  | 5a |  | Extent to which interventions were actually delivered by providers and taken up by participants as planned | Completed in full. |
|  | 5b |  | Where other informational materials about delivering the intervention can be accessed | All questionnaires can be found in supplemental materials. |
|  | 5c |  | When applicable, how intervention providers were assigned to each group | Participants were randomly assigned to a group in the data management platform Qualtrics. |
| Outcomes | 6a | Completely defined pre-specified outcomes, including how and when they were assessed^§^ | Outcomes | **Eligibility Screening.**  Basic Demographics: Participants will confirm their employment as UCSF employees, report their age, gender, assigned sex at birth, and height and weight.  Inclusion and exclusion criteria: Participants will be asked questions relating to our inclusion and exclusion criteria to ensure that they are eligible for the study.  Perceived Stress: The Perceived Stress Scale (PSS) is a gold standard measure for stress perceptions, including ratings of feeling overwhelmed, out of control, and stressed, and has been extensively validated (Cohen et al, 1988). (10 items).  **Baseline Questionnaire Battery:**  Socio-Demographics: household income, level of education, race/ethnicity, job position, UCSF campus location, as well as zip code and phone number for communication purposes. Participants will be given the option to opt-out of text messages.  **Domain: Distress**  Perceived Stress: The Perceived Stress Scale (PSS) is a gold standard measure for stress perceptions, including ratings of feeling overwhelmed, out of control, and stressed, and has been extensively validated (Cohen et al, 1988). (10 items).  Depressive symptoms: The Patient Health Questionnaire-9 (PHQ-9) is a nine-question multipurpose questionnaire to assess symptoms of depression and distress (Kroenke, Spitzer & Williams, 2001) (9 items)  Anxiety: Symptoms of anxiety will be assessed using the Generalized Anxiety Disorder-7 (Spitzer, Kroenke, Williams, & Löwe, 2006), which is a self-reported questionnaire and is routinely used in research (7 items).  Affect: Positive and negative affect will be measured by an 18-item self-report scale (Usala & Hertzog, 1989) that administers an adjective rating scale instrument measuring multiple affective states. Subjects are asked to rate the extent to which each adjective reflected their current mood on a 5-point Likert Scale.  Adverse Childhood Experiences: History of trauma will be evaluated using the Adverse Childhood Experiences Questionnaire, a 10-item survey asking participants to report whether or not they have experienced exposure to abuse or household dysfunction during the first 18 years of life (Felitti et al., 1998).  **Domain: Non-homeostatic eating behaviors**  Reward-Based Eating: The Reward-based Eating Drive (RED-9) scale is a 9-item self-report measure of reward-driven eating and captures a lack of satiety, preoccupation with eating, and loss of control over eating (Epel et al., 2014) (9 items).  USDA 1-item Food Insecurity Worry item: This is a 1-item measure that assesses the extent to which an individual experience worries over running out of food (1 item).  U.S. Household Food Security Module: The six-item short form of the survey module and the associated Six-Item Food Security Scale were developed by researchers at the National Center for Health Statistics in collaboration with Abt Associates Inc. and documented in “The effectiveness of a short form of the household food security scale,” by S.J. Blumberg, K. Bialostosky, W.L. Hamilton, and R.R. Briefel (published by the American Journal of Public Health, vol. 89, pp. 1231-34, 1999). ERS conducted additional assessment of classification sensitivity, specificity, and bias relative to the 18-item scale.    The Sub-study will include the additional measures related to Eating:    *Stress eating question*(Epel, et al., 2004).We will assess *stress eating tendency*using the following 2 items: 1. How do you tend to eat on days when you feel moderately stressed? 2. How do you tend to eat on days when you feel extremely stressed? Item response choices range from *much less than usual* to *much more than usual*.    Questionnaire on Eating and Weight Patterns –5 (QEWP-5). The QEWP-5 (Yanovski, Marcus, Wadden, & Walsh, 2015) is a 24-item questionnaire that assesses frequency of reported binge eating. The QEWP-5 been adapted to capture loss of control (LOC) eating as well as binge episodes. This questionnaire is a screening tool designed to identify adults with possible DSM-5 (American Psychiatric Association, 2013) bulimia nervosa and binge eating disorder.    *Palatable Eating Motives: Coping Subscale (PEMS)(Burgess, Turan, Lokken, Morse, & Boggiano, 2014).*The PEMS Coping subscale is comprised of 4 Likert-like five-choice frequency response items that probe various motives for ‘‘eating tasty food and drinks.’’ The instructions include a list of examples of these kinds of foods and sugary drinks. The list of tasty foods was adopted from the Yale Food Addiction Scale (Gearhardt, Corbin, & Brownell, 2009) with slight modifications. The Coping motives subscale measures intentionally using palatable food to cope with negative feelings (e.g., to forget about or help with worry, depression, nervousness, a bad mood, or problems). Item response choices range from *almost never/never*to*almost always/always.*The trait form for this subscale has been validated using ecological momentary assessment, and both versions are related equally to BMI and changes in emotional eating correlate with changes in BMI over time (Boggiano, Wenger, Turan, Tatum, Morgan, et al., 2015; Boggiano, Wenger, Turan, Tatum, Sylvester, et al., 2015).  *Trait Food Craving Questionnaire, Reduced (FCQ-T-r)(Meule, Hermann, & Kubler, 2014).*The Trait Food Craving Questionnaire, reduced, is a 15-item measure of behavioral, cognitive, and physical aspects of cravings for different types of food. Item response choices range from 0 (*never or n/a*) to 5 (*always*), and the total score is an average of the 15 items. High scores predict how much people crave and eat densely caloric snacks in daily life, and are associated with self-reported failures in dieting (Meule, Lutz, Vogele, & Kubler, 2012; Richard, Meule, Reichenberger, & Blechert, 2017)  Using Food to Cope: this a 2-item questionnaire asking respondents to indicate how they “usually experience a stressful event.” Questions include:  “I eat more of my favorite foods to make myself feel better” and I eat more than I usually do.” Item response choices range from 1 ( a lot) to 4 (not at all). The responses to the items are reverse coded and summed so that higher scores indicate a greater use of food to cope with stress (Tsenkova, Boylan, & Ryff, 2013).    The Reward Based Eating Drive Scale (RED-13) (Mason, et al., 2017): The RED-13 is a 13-item self-report measure that broadly captures the spectrum of reward-related eating (RRE) and may be a useful tool for identifying individuals at risk for overweight or obesity. The RED-13 is designed to capture three dimensions of the RRE construct: lack of control over eating, lack of satiety, and preoccupation with food. RED-13 was also related to self-reported diagnosis of type 2 diabetes as well as cravings for sweet and savory foods (Mason, et al., 2017). Item response choices range from 0 (strongly disagree) to 4 (strongly agree).    Loss of Control over Eating Scale (LOCES) (Latner et al., 2014): The LOCES scale is a multidimensional scale designed to assess LOC eating in both clinical and nonclinical populations (Latner et al. 2014).  Instructions include to ask participants to indicate how often during the past 28 days they have had the following experiences while eating. Each of the 7 questions has a 5- point scale from 1 (Never), 2 (Rarely), 3 (Occasionally), 4 (Often), to 5 (Always)    Food Acceptance and Awareness Questionnaire (FAAQ) (Juarascio, Forman, Timko, Butryn, & Goodwin, 2011):  The FAAQ was designed to measure the acceptance or ability to regulate eating despite urges and cravings and the desire to control these thoughts. The survey is comprised of 10 items and can be answered using a 7-point likert rating scale ranging from 1 (never true) to 7 (always true). Higher scores indicate greater acceptance of motivations to eat (Juarascio et al., 2011).    Food Frequency Questionnaire: (FFQ; modified from the Multi-ethnic Study of Atherosclerosis) ([**Bild et al., 2002**](applewebdata://E7DEBE48-C5A2-4351-BA09-29AB44B832D6#_ENREF_4)), a 16-item self-report measure that assesses how frequently an individual eats foods from the following 16 food categories: processed meats, refined grains, whole grains, fruits, vegetables, white potatoes, fried foods, red meat, high-fat dairy, low-fat dairy, nuts/seeds/peanut butter, fish/seafood (not fried), sodas (non-diet), coffee, sweets, and alcohol. Items for each food group are answered on a Likert scale from 1 (rarely or never) to 9 (two times or more per day) in response to the question: How often do you eat this type of food? Individual scores for each food item are calculated as the frequency, ranging from 1 to 9.  **Domain: Mindfulness**  Mindfulness: This will be measured using the Mindful Attention Awareness Scale (MAAS) (Brown & Ryan, 2003), which assesses the tendency to be mindful day to day (15 items).  Mindwandering probes: We created for this study, a two item measure that assesses present mindwandering and the affective content of this mindwandering (attached).  **Domain: Health Behaviors**  Sleep Quality: overall sleep quality will be assessed using items from the Pittsburgh Sleep Quality Index (Buysse et al., 1989) to assess sleep onset latency, efficiency, duration, and subjective quality (5 items)  Physical activity: The Stanford Leisure-Time Activity (L-Cat) measures usual physical activity habits. We will use a 1-item measure to assess leisure physical activity (Kiernan et al., 2013) (1 item).  **Domain: Physical Health**  Self-reported health: This will be measured using the 12-item Short Form Survey (SF-12), which includes items related to mental and physical well-being and is consistently used in health research (12 items)  Stress-related somatic complaints:  This measure was created for this study (attached) and asks participants to report on the extent they experience the following symptoms: body/joint pain, headaches, stomachaches, nausea, and cold/flu symptoms (5 items).  Checklist of medical and medication history: participants will complete a short check list on common medical conditions and medications they may or may not endorse.  **Domain: Work**  Work Engagement: Work engagement will be assessed using the 9 item self-report Utrech Work Engagement Scale (Schaufeli & Bakker 2003) which uses three factors to determine the level of work engagement: vigor, dedication and absorption.  Days of Work Missed: A two item self-report scale will measure the number of days participants have missed work due to either illness or non-illness related reasons.  Burnout: The Bergen Burnout Inventory is a 9-item measure that assesses three components associated with burnout, including exhaustion, cynicism at work, and sense of inadequacy at work (9 items).  Job Strain: The effort-reward imbalance scale will be used to assess job strain. It contains several subscales that measure effort, reward, and overcommitment and is used routinely in both research and occupational settings (Siegrist et al., 2004) (22 items).  **Midpoint Questionnaire (week 4):**  Mindfulness: This will be measured using the Mindful Attention Awareness Scale (MAAS) (Brown & Ryan, 2003), which assesses the tendency to be mindful day to day (15 items).  Perceived Stress: The Perceived Stress Scale (PSS) is a gold standard measure for stress perceptions, including ratings of feeling overwhelmed, out of control, and stressed, and has been extensively validated (Cohen et al, 1988). (10 items).  Job Strain: The effort-reward imbalance scale will be used to assess job strain. It contains several subscales that measure effort, reward, and overcommitment and is used routinely in both research and occupational settings (Siegrist et al., 2004) (22 items).  Work Engagement: Work engagement will be assessed using the 9 item self-report Utrech Work Engagement Scale (Schaufeli & Bakker 2003) which uses three factors to determine the level of work engagement: vigor, dedication and absorption.  The sub-study participants will also answer the following:  Palatable Eating Motives: Coping Subscale (PEMS)(Burgess, Turan, Lokken, Morse, & Boggiano, 2014). The PEMS Coping subscale is comprised of 4 Likert-like five-choice frequency response items that probe various motives for ‘‘eating tasty food and drinks.’’ The instructions include a list of examples of these kinds of foods and sugary drinks. The list of tasty foods was adopted from the Yale Food Addiction Scale (Gearhardt, Corbin, & Brownell, 2009) with slight modifications. The Coping motives subscale measures intentionally using palatable food to cope with negative feelings (e.g., to forget about or help with worry, depression, nervousness, a bad mood, or problems). Item response choices range from almost never/never toalmost always/always. The trait form for this subscale has been validated using ecological momentary assessment, and both versions are related equally to BMI and changes in emotional eating correlate with changes in BMI over time (Boggiano, Wenger, Turan, Tatum, Morgan, et al., 2015; Boggiano, Wenger, Turan, Tatum, Sylvester, et al., 2015).  **Post intervention Questionnaire (week 8):**  Participants will complete all the same questionnaire included in the baseline packet, with the exception of the medical and medication checklist and Adverse Childhood Events questionnaire. They will be asked about the frequency of their sitting meditation practice in the past 8 weeks.  **Follow-up Questionnaire (2-months post intervention):**  Mindfulness: This will be measured using the Mindful Attention Awareness Scale (MAAS) (Brown & Ryan, 2003), which assesses the tendency to be mindful day to day (15 items).  Perceived Stress: The Perceived Stress Scale (PSS) is a gold standard measure for stress perceptions, including ratings of feeling overwhelmed, out of control, and stressed, and has been extensively validated (Cohen et al, 1988). (10 items).  Job Strain: The effort-reward imbalance scale will be used to assess job strain. It contains several subscales that measure effort, reward, and overcommitment and is used routinely in both research and occupational settings (Siegrist et al., 2004) (22 items).  Work Engagement: Work engagement will be assessed using the 9 item self-report Utrech Work Engagement Scale (Schaufeli & Bakker 2003) which uses three factors to determine the level of work engagement: vigor, dedication and absorption.  The sub-study participants will also answer the following:  Palatable Eating Motives: Coping Subscale (PEMS)(Burgess, Turan, Lokken, Morse, & Boggiano, 2014). The PEMS Coping subscale is comprised of 4 Likert-like five-choice frequency response items that probe various motives for ‘‘eating tasty food and drinks.’’ The instructions include a list of examples of these kinds of foods and sugary drinks. The list of tasty foods was adopted from the Yale Food Addiction Scale (Gearhardt, Corbin, & Brownell, 2009) with slight modifications. The Coping motives subscale measures intentionally using palatable food to cope with negative feelings (e.g., to forget about or help with worry, depression, nervousness, a bad mood, or problems). Item response choices range from almost never/never toalmost always/always. The trait form for this subscale has been validated using ecological momentary assessment, and both versions are related equally to BMI and changes in emotional eating correlate with changes in BMI over time (Boggiano, Wenger, Turan, Tatum, Morgan, et al., 2015; Boggiano, Wenger, Turan, Tatum, Sylvester, et al., 2015).  **Follow-up Questionniare (10-months post intervention; digital meditation condition only):**  Mindfulness: This will be measured using the Mindful Attention Awareness Scale (MAAS) (Brown & Ryan, 2003), which assesses the tendency to be mindful day to day (15 items).  Perceived Stress: The Perceived Stress Scale (PSS) is a gold standard measure for stress perceptions, including ratings of feeling overwhelmed, out of control, and stressed, and has been extensively validated (Cohen et al, 1988). (10 items).  Job Strain: The effort-reward imbalance scale will be used to assess job strain. It contains several subscales that measure effort, reward, and overcommitment and is used routinely in both research and occupational settings (Siegrist et al., 2004) (22 items).  Work Engagement: Work engagement will be assessed using the 9 item self-report Utrech Work Engagement Scale (Schaufeli & Bakker 2003) which uses three factors to determine the level of work engagement: vigor, dedication and absorption.  Participants may also complete the following:  U.S. Household Food Security Module: The six-item short form of the survey module and the associated Six-Item Food Security Scale were developed by researchers at the National Center for Health Statistics in collaboration with Abt Associates Inc. and documented in “The effectiveness of a short form of the household food security scale,” by S.J. Blumberg, K. Bialostosky, W.L. Hamilton, and R.R. Briefel (published by the American Journal of Public Health, vol. 89, pp. 1231-34, 1999). ERS conducted additional assessment of classification sensitivity, specificity, and bias relative to the 18-item scale.  Participants were assured that their survey responses would not be shared with their supervisors or other University personnel and would not be associated with any other University records. The study was approved by the Committee on Human Research (CHR) at UCSF, and all participants signed a written consent for their participation.    Substudy:  The sub-study participants will also answer the following:  Palatable Eating Motives: Coping Subscale (PEMS)(Burgess, Turan, Lokken, Morse, & Boggiano, 2014). The PEMS Coping subscale is comprised of 4 Likert-like five-choice frequency response items that probe various motives for ‘‘eating tasty food and drinks.’’ The instructions include a list of examples of these kinds of foods and sugary drinks. The list of tasty foods was adopted from the Yale Food Addiction Scale (Gearhardt, Corbin, & Brownell, 2009) with slight modifications. The Coping motives subscale measures intentionally using palatable food to cope with negative feelings (e.g., to forget about or help with worry, depression, nervousness, a bad mood, or problems). Item response choices range from almost never/never toalmost always/always. The trait form for this subscale has been validated using ecological momentary assessment, and both versions are related equally to BMI and changes in emotional eating correlate with changes in BMI over time (Boggiano, Wenger, Turan, Tatum, Morgan, et al., 2015; Boggiano, Wenger, Turan, Tatum, Sylvester, et al., 2015).  Anthropometric measurements included weight measurement using a digital scale, height using a stadiometer, waist and hip circumference using a cloth tape measure, and sagittal diameter using an anthropometer measuring stick device.  Participants had a fasting blood draw using a finger stick, which were immediately processed into serum, plasma, and whole blood aliquots and frozen at -80 C for batch assay by the research laboratory of Dr. Peter Havel at the University of California, Davis. Fasting blood samples were assayed for glucose, insulin, hemoglobin A1c (HbA1c), lipid profile (including cholesterol, low density lipoproteins or LDL, high density lipoproteins or HDL, triglycerides, ApoA1, and ApoB lipoproteins), uric acid, liver enzymes (gamma-glutamyl transpeptidase (GGT) and alanine transaminase (ALT). |
|  | 6b | Any changes to trial outcomes after the trial commenced, with reasons |  | No changes. |
| Sample size | 7a | How sample size was determined^§^ | Sample size | We will screen a high number of potential participants to carry out this study. In this regard, we will screen as many as 10000 individuals and thus as many as 10000 individuals will complete a consent form to be screened. Of those who complete the screening, we anticipate that we will consent into the study 2000 participants.  We will randomize 1000 participants to our digital mediation condition (Headspace) and 1000 to the waitlist control condition.  Substudy:  We are interested in testing a digital intervention with limited contact by study investigators (i.e., no in person contact). As such, participant attrition is to be expected.  Our prior study (Bostock et al., under revision) detected effects in a sample of < 250 participants.  We expect that our sample size will be well powered to detect improvements in our self-report measures in response to our treatment intervention. |
|  | 7b | When applicable, explanation of any interim analyses and stopping guidelines |  | N/A |
| Randomisation |  |  |  |  |
| Sequence generation | 8a | Method used to generate the random allocation sequence |  | The randomization for the larger study was performed by the computer-generated program Qualtrics once the participant completed the baseline questionnaire.  Substudy:  Once the baseline assessment was completed, the participant was randomly assigned to one of four groups on Qualtrics. |
|  | 8b | Type of randomisation and details of any restriction (such as blocking and block size)^§^ |  | The randomization was performed by the research assistant by using a computer-generated program Qualtrics for randomizing people to condition 1 (intervention) or 2 (waitlist). The program insures even distribution. Participants were emailed their randomization status. |
| Allocation concealment mechanism | 9 | Mechanism used to implement the random allocation sequence, describing any steps taken to conceal the sequence until interventions were assigned^§^ |  | Once the participant completed the baseline questionnaire, they were randomly assigned to the waitlist or intervention group.  Substudy: When a new participant was ready to be randomized, study personnel used a survey created on Qualtrics and inputted the participants email, phone number, and participant ID. After the survey was completed, participants were randomly assigned to one of four groups.  Study personnel were not able to access the file containing the sequence of assignments or to see the next condition in the sequence until the moment they randomized the participant. |
| Implementation | 10 | Who generated the random allocation sequence, who enrolled participants, and who assigned participants to interventions^§^ |  | The sequence of assignments was generated ahead of time with a computer script by a statistician who was not involved in running the study. |
| Awareness of assignment | 11a | Who was aware of intervention assignment after allocation (for example, participants, providers, those assessing outcomes), and how any masking was done |  | Both participants and study staff were unblinded to the assignment after allocation. |
|  | 11b | If relevant, description of the similarity of interventions |  |  |
| Analytical methods | 12a | Statistical methods used to compare group outcomes^§^ | How missing data were handled, with details of any imputation method |  |
|  | 12b | Methods for additional analyses, such as subgroup analyses, adjusted analyses, and process evaluations |  |  |
| Results |  |  |  |  |
| Participant flow (a diagram is strongly recommended) | 13a | For each group, the numbers randomly assigned, receiving the intended intervention, and analysed for the outcomes^§^ | Where possible, the number approached, screened, and eligible prior to random assignment, with reasons for non-enrolment |  |
|  | 13b | For each group, losses and exclusions after randomisation, together with reasons^§^ |  |  |
| Recruitment | 14a | Dates defining the periods of recruitment and follow-up |  | Study start date: 2/14/2019  Primary completion date: 5/19/2020  Study completion date: 3/1/2021 |
|  | 14b | Why the trial ended or was stopped |  | Recruitment ended when we met our goal of 150 participants. |
| Baseline data | 15 | A table showing baseline characteristics for each group^§^ | Include socioeconomic variables where applicable |  |
| Numbers analysed | 16 | For each group, number included in each analysis and whether the analysis was by original assigned groups^§^ |  |  |
| Outcomes and estimation | 17a | For each outcome, results for each group, and the estimated effect size and its precision (such as 95% confidence interval)^§^ | Indicate availability of trial data |  |
|  | 17b | For binary outcomes, presentation of both absolute and relative effect sizes is recommended |  |  |
| Ancillary analyses | 18 | Results of any other analyses performed, including subgroup analyses, adjusted analyses, and process evaluations, distinguishing pre-specified from exploratory |  |  |
| Harms | 19 | All important harms or unintended effects in each group (for specific guidance, see CONSORT for Harms) |  | One participant experienced a small skin infection after undergoing the finger stick glucose assessment during their baseline visit. |
| Discussion |  |  |  |  |
| Limitations | 20 | Trial limitations, addressing sources of potential bias, imprecision, and, if relevant, multiplicity of analyses |  |  |
| Generalisability | 21 | Generalisability (external validity, applicability) of the trial findings^§^ |  |  |
| Interpretation | 22 | Interpretation consistent with results, balancing benefits and harms, and considering other relevant evidence |  |  |
| Important information |  |  |  |  |
| Registration | 23 | Registration number and name of trial registry |  | Substudy: NCT03945214 |
| Protocol | 24 | Where the full trial protocol can be accessed, if available |  |  |
| Declaration of interests | 25 | Sources of funding and other support, role of funders | Declaration of any other potential interests | This study was supported by a research grant from Headspace, Inc and UCOP Healthy Campus Network. The funders had no role in the design, conduct, analysis and reporting of the trial. |
| Stakeholder involvement | 26a |  | Any involvement of the intervention developer in the design, conduct, analysis, or reporting of the trial | The same team of researchers designed, conducted analysis and reported on the trail. |
|  | 26b |  | Other stakeholder involvement in trial design, conduct, or analyses |  |
|  | 26c |  | Incentives offered as part of the trial | Employee participants who completed the study received a free year subscription to Headspace were entered in a raffle for a trip to Napa, CA (a value of $1000).  Sub study:  Participants who completed the study received a free year subscription to Headspace, a Fitbit, and were entered in a raffle for a trio to Napa, CA (a value of $1000). |
| This table lists items from the CONSORT 2010 checklist (with some modifications for social and psychological intervention trials as described in Table [​Table2)2](https://www.ncbi.nlm.nih.gov/pmc/articles/PMC6066913/table/Tab2/)) and additional items in the CONSORT-SPI 2018 extension. Empty rows in the ‘CONSORT-SPI 2018’ column indicate that there is no extension to the CONSORT 2010 item | | | | |
