# Supplementary material for: Impact of digital meditation on work stress and health outcomes among adults with overweight: A randomized controlled trial
Source: PLoS One. 2023 Mar 1;18(3):e0280808. doi: 10.1371/journal.pone.0280808 (PMC9977041; doi:10.1371/journal.pone.0280808)
Supplement: S2 File — (PDF) [file pone.0280808.s003.pdf]

Study Application (Version 1.12)

1.0 General Information

***Enter the full title of your study:**

Stress Free UCSF

***Enter the study number or study alias**

Stress Free UC
* This field allows you to enter an abbreviated version of the Study Title to quickly identify this study.

2.0 Add Department(s)

2.1 List departments and/or research programs associated with this study:

Primary Dept?	Department Name
(•)	**UCSF** - *133100* - M_Psychiatry

3.0 List the key study personnel: (Note: external and affiliated collaborators who are not in the UCSF directory can be identified later in the Qualifications of Key Study Personnel section at the end of the form)

3.1 *Please add a Principal Investigator for the study:

Aric A Prather, PhD

Select if applicable

- [ ] Department Chair
- [ ] Resident
- [ ] Fellow

If the Principal Investigator is a Fellow, the name of the Faculty Advisor must be supplied below.

3.2 If applicable, please select the Research Staff personnel:

A) Additional Investigators

Crosswell, Alexandra D
Other Investigator
Epel, Elissa Ph.D.
Co-Principal Investigator
Radin, Rachel M
Other Investigator

B) Research Support Staff

Fisher, Sarah M
Study Coordinator
Fromer, Elena N
Research Assistant
Schilf, Samantha
Study Coordinator
Vaccaro, Julie M
Study Coordinator

3.3 *Please add a Study Contact:

Schilf, Samantha
Vaccaro, Julie M

The Study Contact(s) will receive all important system notifications along with the Principal Investigator. (e.g. The project contact(s) are typically either the Study Coordinator or the Principal Investigator themselves).

3.4 If applicable, please add a Faculty Advisor/Mentor:

3.5 If applicable, please select the Designated Department Approval(s):

Add the name of the individual authorized to approve and sign off on this protocol from your Department (e.g. the Department Chair or Dean).

4.0 Initial Screening Questions

Updated January 2019 - Revised Common Rule (January 2018) Compliant - v92

4.1 * PROJECT SUMMARY: (REQUIRED) Give a brief overview of this project (250 words or less). Tell us what this study is about, who is being studied, and what it aims to achieve. If you have an NIH Abstract, paste it here (Click on the orange question mark to the right for more detailed instructions):

The aim of the present study is to test the effects of a digital meditation intervention in a sample of high stress UCSF employees. We propose to randomize 1000 UCSF employees to 8-weeks of either a digital mindfulness intervention (using the commerically available application Headspace) or a waitlist control condition. Measurement time points include baseline, week 4, week 8 (post intervention), and 4-month follow up period. Among participants who are randomized to the digital meditation intervention, they will also take part in a 1-year follow up. Our primary outcomes will be reductions in global levels of perceived stress and job strain. Secondary outcomes include improvements in mindfulness, symptoms of anxiety and depression, health behaviors, including sleep, physical activity, and eating behavior, and health care utilization.

Within this larger study, we will also conduct a smaller more focused substudy in which we will recruit over weight and obese participants (BMI => 25). This study will include all of the measures obtained in the larger, main study but also expand to include physiological assessments, personal fitness and sleep metrics, and measures of daily mood data. The physiological assessments will take place at baseline and week 8, and the fitness, sleep, and mood data will be gathered for seven consecutive days at baseline and week 7. All other measurement time points are similar to those of the main study. For this substudy, we will recruit 150 additional UCSF employees, who will be randomly assigned to one of four conditions: digital meditation, a healthy eating intervention, digital meditation+healthy eating intervention, or a waitlist control condition. If participants are assigned to either of the Healthy Eating conditions, in addition

to the above-mentioned assessments, they will participate in one 30- to 60-minute counseling session centered around healthy eating behaviors such as decreasing sugar intake or the frequency of stress-related eating, and three 10-minute booster phone calls at week 1, week 4, and week 8.

4.2 * HUD DEVICE: (REQUIRED) Does this application involve a Humanitarian Use Device (HUD):

- (•) No
- ( ) Yes, and it includes a research component
- ( ) Yes, and it involves clinical care ONLY

4.3 * TYPE OF RESEARCH: (REQUIRED) Select the option that best fits your project (Click the orange question mark to the right for definitions and guidance):

- ( ) Biomedical research (including medical records review, biospecimen collection and/or use, other healthcare or health outcomes related activities, research database, biospecimen bank, or recruitment registry)
- (•) Social, behavioral, educational, and/or public policy research
- ( ) Hybrid - includes aspects of BOTH types of research (check this option if your research is mainly social /behavioral but also involves specimen collection or blood draws to look at biological measures)

4.4 * SUBJECT CONTACT: (REQUIRED) Does this study involve ANY contact or interactions with participants:

- (•) Yes (including phone, email or web contact)
- ( ) No (limited to medical records review, biological specimen analysis, and/or data analysis)

4.5 * RISK LEVEL: (REQUIRED) What is your estimation of the risk level, including all screening procedures and study activities:

- (•) Minimal risk
- ( ) Greater than minimal risk

4.6 * REVIEW LEVEL: (REQUIRED) Requested review level (Click on the orange question mark to the right for definitions and guidance):

- ( ) Full Committee
- (•) Expedited
- ( ) Exempt

4.7 * EXPEDITED REVIEW CATEGORIES: (REQUIRED) If you think this study qualifies for expedited review, select the regulatory categories that the research falls under: (check all that apply)

- [ ] Category 1: A very limited number of studies of approved drugs and devices
- [x] Category 2: Blood sampling
- [ ] Category 3: Noninvasive specimen collection (e.g. buccal swabs, urine, hair and nail clippings, etc.)
- [ ] Category 4: Noninvasive clinical procedures (e.g. physical sensors such as pulse oximeters, MRI, EKG, EEG, ultrasound, moderate exercise testing, etc.)
- [x] Category 5: Research involving materials (data, documents, records, or specimens) that were previously collected for either nonresearch or research purposes
- [ ] Category 6: Use of recordings (voice, video, digital or image)
- [x] Category 7: Low risk behavioral research or research employing survey, interview, oral history, focus group, program evaluation, human factors evaluation, or quality assurance methodologies

* Does the collection of blood samples meet requirements outlined by HHS Office for Human Research Protections for **Expedited Review Research Category 2**: **(REQUIRED)**

- For healthy, nonpregnant adults who weigh at least 110 pounds the amounts drawn may not exceed 550 ml in an 8 week period and collection may not occur more frequently than 2 times per week;
- From other adults and children, considering the age, weight, and health of the subjects, the collection procedure, the amount of blood to be collected, and the frequency with which it will be collected, the amount drawn may not exceed the lesser of 50 ml or 3 ml per kg in an 8 week period and collection may not occur more frequently than 2 times per week

(•) Yes ( ) No

4.9 *** DATA/SPECIMEN ANALYSIS ONLY: (REQUIRED) Does this study ONLY involve records review and /or biospecimen analysis (do not check 'Yes' if this is a registry, research or recruitment database, or biospecimen repository):**

( ) Yes (•) No

4.10 *** CLINICAL TRIAL: (REQUIRED)**
Is this a clinical trial:

According to The World Health Organization (WHO) and the International Committee of Medical Journal Editors (ICMJE) a clinical trial is:

- Any research study that prospectively assigns human participants or groups of humans to one or more health-related interventions to evaluate the effects on health outcomes.

ICMJE requires registration of a clinical trial in a public database (such as ClinicalTrials.gov) prior to enrollment, for eventual publication of results in member biomedical journals.
Guidance: Public Law 110-85 requires that all investigators who perform an *applicable clinical trial* must ensure that the trial is registered on a government web site called **ClinicalTrials.gov**.

The FDA requires registration for 'applicable clinical trials,' defined as follows:

- For any trials of drugs and biologics: controlled clinical investigations, other than Phase 1 investigations, of a product subject to FDA regulation.
- For trials of biomedical devices: controlled trials with health outcomes of devices subject to FDA regulation, other than small feasibility studies, and pediatric post-market surveillance.

For additional information on the **ClinicalTrials.gov** registration process at UCSF and the definition of a clinical trial for purposes of registration, visit the **ClinicalTrials.gov section of the UCSF Clinical Research Resource HUB**.

(•) Yes ( ) No

Clinical Trial Registration - 'NCT' number for this trial:

NCT03527303

4.11 * CLINICAL TRIAL PHASE: (REQUIRED) Check the applicable phase(s):

- [ ] Phase 0
- [ ] Phase 1
- [ ] Phase 1/2
- [ ] Phase 2
- [ ] Phase 2/3
- [x] Phase 3
- [ ] Phase 4
- [ ] Not Applicable

4.12 * INVESTIGATOR-INITIATED: (REQUIRED) Is this an investigator-initiated study:

(•) Yes ( ) No

The UCSF IRB recommends use of the Virtual Regulatory Binder to manage your study.

4.13 * CANCER: (REQUIRED) Does this study involve cancer (e.g., the study involves patients with cancer or at risk for cancer, including behavioral research, epidemiological research, public policy research, specimen analysis, and chart reviews):

( ) Yes (•) No

4.14 * RADIATION EXPOSURE: (REQUIRED) Does your protocol involve any radiation exposure to patients /subjects EITHER from standard care OR for research purposes (e.g., x-rays, CT-scans, DEXA, CT-guided biopsy, radiation therapy, or nuclear medicine including PET, MUGA or bone scans):

( ) Yes (•) No

4.15 SCIENTIFIC REVIEW: If this study has undergone scientific or scholarly review, please indicate which entity performed the review (check all that apply):

- [ ] Cancer Center Protocol Review Committee (PRC) (Full approval is required prior to final IRB approval for cancer-related protocols.)
- [ ] CTSI Clinical Research Services (CRS) Advisory Committee
- [ ] CTSI Consultation Services
- [ ] Departmental scientific review
- [x] Other:

* Specify **Other: (REQUIRED)**

UCSF Healthy Campus Network

4.16 * STEM CELLS: (REQUIRED) Does this study involve human stem cells (including iPS cells and adult stem cells), gametes or embryos:

(•) No
( ) Yes, and requires IRB and GESCR review
( ) Yes, and requires GESCR review, but NOT IRB review

4.17 *** FINANCIAL INTERESTS: (REQUIRED) Do you or any other responsible personnel (or the spouse, registered domestic partner and/or dependent children thereof) have financial interests related to this study:**

○ Yes ◉ No

5.0 Funding

5.1 *** FEDERAL FUNDING: (REQUIRED) Is this study currently supported in whole or in part by Federal funding, *even by a subcontract*, OR has it received ANY Federal funding in the past:**

○ Yes ◉ No

5.2 *** DoD INVOLVEMENT: Is this project linked in any way to the Department of Defense (DoD): (REQUIRED)**

○ Yes ◉ No

5.3 **SPONSORS: Identify all sponsors and provide the funding details. If funding comes from a Subcontract, please list only the Prime Sponsor:**

External Sponsors:

View Details	Sponsor Name	Sponsor Type	Awardee Institution	Contract Type:	UCSF RAS "P number" or eProposal number	UCSF RAS System Award Number ("A" + 6 digits)
⊟	Headspace, Inc	04	UCSF	Contract	P0528111	A130873

Sponsor Name:	Headspace, Inc
Sponsor Type:	04
Sponsor Role:	Funding
**Grant/Contract Number:**	N/A
Awardee Institution:	UCSF
**Is Institution the Primary Grant Holder:**	Yes
Contract Type:	Contract
UCSF RAS "P number" or eProposal number:	P0528111
UCSF RAS System Award Number ("A" + 6 digits):	A130873
Grant Number for Studies Not Funded thru UCSF:	
Grant Title:	Impact of 8 weeks of Headspace on stress in a heterogeneous university employee cohort (Protocol HS-17-03)
PI Name:<br>(If PI is not the same as identified on the study.)	
Significant Discrepancy:	

Other Funding Sources and Unfunded Research - Gift, Program, Departmental or other Internal Funding (check all that apply):

☐ Funded by gift (specify source below)
☑ Funded by UCSF or UC-wide program (specify source below)
☐ Specific departmental funding (specify source below)
☐ Unfunded (miscellaneous departmental funding)
☐ Unfunded student project

* Identify the gift, program, departmental, or other internal funding source: **(REQUIRED)**

UCOP funded Healthy Campus Network

6.0 Sites, Programs, Resources, and External IRB Review

6.1 UCSF AND AFFILIATED SITES (check all that apply):

☐ UCSF Benioff Children's Hospital Oakland (BCHO)
☐ UCSF China Basin clinics and facilities
☐ UCSF Helen Diller Family Comprehensive Cancer Center
☑ UCSF Langley Porter Psychiatric Institute (LPPI)
☑ UCSF Medical Center at Mission Bay (Benioff Children's Hospital, the Betty Irene Moore Women's Hospital, Bakar Cancer Hospital, or outpatient clinics)
☑ UCSF Mount Zion
☑ UCSF Parnassus (Moffitt-Long hospital, dental clinics or other outpatient clinics)
☑ UCSF Other Sites (including Laurel Heights and all the other sites outside the main hospitals)
☐ Zuckerberg San Francisco General (ZSFG)
☐ SF VA Medical Center (SF VAMC)
☐ Fresno - UCSF Fresno OR Community Medical Center (CMC)
☐ Gladstone
☐ Institute on Aging (IOA)
☐ Jewish Home
☐ SF Dept of Public Health (DPH)
☐ Vitalant (formerly Blood Centers of the Pacific and Blood Systems Research Institute)

6.2 LOCATIONS: At what locations will study visits and activities occur:

All activities- screening, registration, surveys, etc., will take place online. Participants can access questionnaires, assessments, and all other study materials on their own time on a computer or smartphone.

Sub-study: Screening, surveys, and digital components of the intervention will take place online. The in-person clinic visits at baseline and week 8 will take place at the Parnassus campus at Langley Porter Psychiatric Institute. Counseling sessions will be scheduled in a private room at the UCSF campus at which the participant works.

6.3 OFF-SITE PROCEDURES: Will any study procedures or tests be conducted off-site by non-UCSF personnel:

○ Yes ● No

6.4 RESEARCH PROGRAMS: Check any UCSF research programs this study is associated with:

☐ Cancer Center

☐ Center for AIDS Prevention Sciences (CAPS)
☐ Global Health Sciences
☐ Immune Tolerance Network (ITN)
☐ Neurosciences Clinical Research Unit (NCRU)
☐ Osher Center
☐ Positive Health Program

6.5 * CTSI CRS SERVICES: (REQUIRED) Will this study be carried out at one of the UCSF Clinical Research Services (CRS) units or utilize CRS services:

○ Yes ◉ No

6.6 * MULTI-CENTER TRIAL: (REQUIRED) Is this a multi-center or multi-site research trial:

By '**multi-center trial**' we mean a study where the protocol is developed by an lead investigator, an industry sponsor, consortium, a disease-group, etc.,and multiple sites across the nation or in different countries participate in the trial. The local sites do not have any control over the design of the protocol.

○ Yes ◉ No

6.8 OTHER SITE TYPES: Check all the other types of sites not affiliated with UCSF with which you are cooperating or collaborating on this project:

Do NOT check any boxes below if this is a multi-center clinical trial, UCSF is just one of the sites, and neither UCSF nor one of its faculty-linked affiliates (SF VAMC, Gladstone, ZSFG) are the coordinating center.

☐ Other UC Campus

☐ Other institution

☐ Other community-based site

☐ Foreign Country

☐ Sovereign Native American nation (e.g. Navajo Nation, Oglala Sioux Tribe, Havasupai, etc.)

6.14 * RELYING ON AN EXTERNAL IRB: (REQUIRED) Does this application include a request to rely on an external IRB (a central IRB (other than the NCI CIRB) or an external IRB (other UC campus, commercial, or institutional):

○ Yes ◉ No

7.0 Research Plan and Procedures

7.1 HYPOTHESIS: Describe the hypothesis or what the study hopes to prove:

Primary;

Hypothesis 1: Participants randomized to the 8-week digital meditation intervention will report greater reduction in global perceptions of stress and job strain compared to those randomized to the waitlist control condition.

Secondary:

Hypothesis 2: Participants randomized to the 8-week digital meditation intervention will report greater increases in subjective mindfulness, as measured by the Mindful Awareness and Attention Scale, compared to those in the waitlist control condition. The increase in mindfulness will statistically mediate improvements in global stress perception and job strain.

Hypothesis 3: Treatment adherence to the 8-week digital meditation intervention will moderate observed treatment effects, such that improvements in global stress perception and job strain will be stronger among participants who completed more meditations.

Sub-study:

Hypothesis 1. Compared to the Waitlist control condition, participants randomized Digital Meditation, Healthy Eating, or Digital Mediation+Healthy Eating will show greater decreases in blood pressure, improvements in metabolic outcomes, and improvements in measures of psychological stress. Effects will be strongest in the Digital Meditation+Healthy Eating condition.

7.2 AIMS: List the specific aims:

Aim 1: To test the effects of a digital meditation intervention, compared to a waitlist control condition, on global perceptions of stress and job strain in a sample of UCSF employees.

Aim 2: To identify whether increases in mindfulness serves as a pathway through which digital meditation affects reports of stress and test whether treatment adherence moderates treatment-related stress effects.

Sub-study

Aim 1. To examine the effect of Digital Meditation, Healthy Eating, and Digital Meditation+Healthy Eating on improvements in blood pressure, markers of metabolic health, and psychological stress in adults who are overweight and reporting moderate stress.

7.3 DESIGN: Briefly describe the study design (e.g., observational, interventional, randomized, placebo-controlled, blinded, cross-over, cross-sectional, longitudinal, pharmacokinetic, etc.):

We propose a randomized controlled trial testing the benefits of an 8-week digital intervention compared to a waitlist control condition in 2,000 high stress (i.e., perceived stress score of 15 or higher) UCSF employees. Eligible participants will complete a baseline assessment and then undergo randomization. Participants randomized to the digital meditation condition will use the smartphone/computer application called Headspace which provides digital audio mediations that are 10 minutes in duration. There will be a midpoint assessment at week 4 of the intervention, a post-intervention assessment at week 8, and a follow-up assessment +4 months from baseline. Those randomized to the waitlist control condition will then be provided access to the digital meditation condition. All questionnaires will be provided electronically and there will be no in person assessments. Among participants initially randomized to the digital meditation condition there will be an additional 1-year follow-up questionniare to assess durability of the intervention.

We propose an additional sample of 150 participants to examine a mindfulness program along with a healthy eating program. Eligible participants (with a BMI of 25 or above) who have consented to this sub-study, will complete online surveys, as well as in-person clinic assessments. At these assessments, study staff will orient participants to the study, collect anthropometric (e.g., weight, waist to hip ratio) and physiological measurements (e.g., fasting glucose) at pre-and post-intervention. Then, subjects will complete the baseline questionnaire online. One week prior to randomization, participants will complete twice-daily surveys in the morning and at night for 7 days, while wearing a fitness watch that collects various measures such as physical activity and sleep. Participants randomized to the digital meditation condition will use the smartphone/computer application called Headspace which provides digital audio mediations that are 10 minutes in duration. Those randomized to the waitlist control condition will then be provided access to the digital meditation condition. Participants may also take part in a healthy eating program designed to assess and reduce problematic eating behavior such as stress-related eating. There will be a midpoint assessment at week 4 of the intervention, a post-intervention assessment at week 8, and a follow-up assessment 4 months from baseline. During week 7 of the intervention, participants will once again be asked to complete the weeklong daily and nightly diaries and wear the fitness watch. At week 8, they will partake in a second in-person clinical visit. Among participants initially randomized to the digital meditation condition there will be an additional 1-year follow-up questionnaire to assess durability of the intervention.

7.4 BACKGROUND AND SIGNIFICANCE: Briefly provide the background and significance of this study (e.g. why is this study needed) (space limit: one half page):

High levels of psychosocial work-related stress have major implications for both the employee and the employer. Epidemiological studies consistently demonstrate associations between high work stress and worse self-reported mental and physical health, including depression, anxiety, cardiovascular disease, and type 2 diabetes (Ganster & Rosen, 2013). In the United States, it's estimated that 5-8% of annual healthcare costs are attributable to work-related stressors (Goh, Pfeffer, & Zenios, 2016). In the United Kingdom, the overall annual cost of work-related stress to employers is estimated to be over £26bn, driven by increased staff turnover, performance degradation, and absenteeism (The Sainsbury Centre for Mental Health, 2007).

Job strain, a combination of high demands (workload and intensity) and low control (discretion over work tasks), is one of the most widely studied models used to define psychosocial stress at work (Karasek, 1979). Epidemiological studies and meta-analyses of decades of research have found that job strain is associated with worse mental and physical health, including anxiety and depressive disorders, increased blood pressure, cardiovascular events, and metabolic syndrome (Chandola, Brunner, & Marmot, 2006; Landsbergis, Dobson, Koutsouras, & Schnall, 2013; Madsen et al., 2017; Steptoe & Kivimäki, 2013). Mechanisms linking job strain to poor physical health include worse health behaviors, obesity, and allostatic load (Chandola, Britton, Brunner, et al., 2008; Ganster & Rosen, 2013; Nyberg et al., 2013).

The potential stress-reduction and psychological well-being benefits of teaching mindfulness in the workplace has received increased attention and initial empirical support (Jamieson & Tuckey, 2017). There are many conceptualizations of mindfulness. Here we define being 'mindful' as being in a state in which one is paying full attention to their present moment experience with openness and non-judgmental acceptance (Kabat-Zinn, 1982). Meta-analyses have reported that mindfulness-based psychological interventions decrease stress in healthy non-clinical populations, and improve psychosocial outcomes for people with clinical disorders such as anxiety and depression (Bohlmeijer, Prenger, Taal, & Cuijpers, 2010; Chiesa & Serretti, 2009; Hofmann, Heering, Sawyer, & Asnaani, 2009; Kuyken, Warren, Taylor, & Whalley, 2016). Mindfulness-based trainings delivered in the workplace have been shown to decrease global perceptions of psychological stress in healthy working adults (Virgili, 2015).

Mechanisms proposed to explain the stress reduction benefits of mindfulness-based therapies include an improved capacity to cope with stressful situations and enhanced attention regulation (Hölzel et al., 2011). Specifically, mindfulness training may promote the positive reappraisal of stressful circumstances as benign or meaningful (Garland, Gaylord, & Park, 2009), and can improve recovery from negative emotional events (Crosswell et al., 2017). In a work context, these enhanced coping abilities may lead to the reappraisal of demands as manageable and work stressors as within one's control, leading to decreased job strain. Good et al. (2016) present an integrated model of the effects of mindfulness training on outcomes relevant for the workplace in which improved attention stability, control, and efficiency lead to improvements in cognitive, emotional, behavioral, and physiologic domains, which ultimately improve job performance, workplace relationships, and well-being. There is initial support for this model from evidence that dispositional mindfulness is positively associated with many of these psychological and social factors, however workplace mindfulness intervention trials have primarily focused on decreasing psychological stress, with limited empirical evidence showing that mindfulness training indeed leads to improvements in the other domains of the model.

Mindfulness training delivered via self-guided smartphone app may offer a convenient alternative to group sessions. App-based treatments to improve mental health are an increasingly popular method of service delivery, though research on the efficacy of these apps is limited (Donker et al., 2013; Fairburn &

Patel, 2017). Initial evidence of technology-driven delivery of standard treatment protocols for clinical disorders such as Cognitive Behavioral Therapy for anxiety have demonstrated effect sizes comparable to conventional standard of care (e.g. Berger, Boettcher, & Caspar, 2014; Titov et al., 2011). Three small studies using smartphone apps to deliver mindfulness interventions to healthy adults found benefits comparable to traditional delivery methods on outcomes of subjective well-being, depressive symptoms, and compassion (Howells, Ivtzan, & Eiroa-Orosa, 2016; Lim, Condon, & De Steno, 2015; Ly et al., 2014). App-based interventions also offer the benefit of standardization of instruction across participants in the experimental group, ability for participants to control where and when they access the intervention, and objective measures of adherence to the intervention via data collected on the app's backend rather than self-report.

The purpose of this study is to examine the effects of a mindfulness meditation program delivered via smartphone on global perceptions of psychological distress and work stress (i.e., job strain) in a sample of healthy employed adults at UCSF with high levels of global stress.

7.5 PRELIMINARY STUDIES: Briefly summarize any preliminary studies relevant to your proposed research (space limit: one half page):

We recently investigated whether a mindfulness meditation program delivered via a smartphone application (app; Headspace) could improve psychological well-being, reduce job strain, and reduce ambulatory blood pressure during the workday. Participants were 238 healthy employees from two large UK companies that were randomized to a wait-list group or a mindfulness meditation practice app. Use of the app was associated with significant improvement in well-being, job strain, and perceptions of workplace social support relative to the wait-list control group. Meditating a greater number of times (i.e., better treatment adherence) was associated with the greatest improvements in psychological outcomes. Participants who completed 25 or more meditation sessions over 8 weeks also had significantly lower self-measured systolic blood pressure over the course of one day compared to the control condition participants at the follow-up time point. Sustained positive effects of the intervention were seen for well-being and job strain 8 weeks after the end of the intervention period. This trial suggests that almost daily brief mindfulness meditations delivered via smartphone can improve outcomes related to workplace stress and well-being, with potentially lasting effects.

This study is presently under revision in a peer-reviewed journal:

Bostock, S., Crosswell, A., Prather, A.A., Steptoe, A. Mindfulness on-the-go: Effects of a mindfulnessmeditation app on work-related stress. *Under revision at Journal of Occupational Health Psychology*

7.6 * TREATMENT PROTOCOL: Is this a treatment study, i.e. does this study intend to provide treatment to individuals with a medical or psychological condition: (REQUIRED)

(•) Yes ( ) No

7.7 * BILLABLE PROCEDURES: Does this study involve any procedures, lab tests or imaging studies that have a CPT code and could be billable to patients, their insurance, Medi-Cal, Medicare, or any other entity (answer 'Yes' even if the study is going to pay for all the procedures): (REQUIRED)

( ) Yes (•) No

If you are not sure if your study involves billable procedures, send an email to the UCSF Office of Clinical Research (OCR) for help answering this question.

7.8 * COMMON RESEARCH ACTIVITIES: Types of research activities that will be carried out. Check all that apply and describe in more detail in the 'Procedures / Methods' section: (REQUIRED)

- [x] Interviews, questionnaires, surveys
- [ ] Educational or cognitive tests
- [ ] Focus groups
- [ ] Social media-based research activities
- [ ] Observation
- [ ] Fitness tests or other exertion activities
- [x] Use of mobile health apps or other apps
- [x] Collection of data from wearable tech such as Fitbit, Apple Watch, Garmin, motion actigraphs, etc.)
- [ ] Non-invasive imaging or testing (MRI, EEG, pulse oximetry, etc.)
- [ ] Imaging procedures or treatment procedures that involve radiation (x-rays, CT scans, CT-guided biopsies, DEXA scans, MUGA or PET scan)
- [ ] Administration of contrast agent
- [ ] Randomization to one intervention versus another
- [ ] Use of placebo
- [ ] Biopsy conducted solely for research purposes
- [ ] Sham surgical procedure
- [ ] None of the above

7.9 * PROCEDURES / METHODS: (REQUIRED)

Describe the research methods and study activities taking place at each site (e.g. what will participants be asked to do and what will members of the study team do?). If there will be multiple participant groups or study sites, explain what will happen with each group or study sites.

If some of the activities would occur even if the person were not in the study, as in the case of treatment or tests performed for diagnostic purposes, **clearly differentiate between those activities that will be done solely for research purposes and those that are happening as part of routine care.**

Please call our office at 415-476-1814 and ask to speak to someone on the Expedited Review team if you need help differentiating between what parts are research and what parts aren't.

Below we list the research methods and study activities:

The main study is designed to be carried out over the internet and to be as automated as possible. Interactions with participants will be facilitated online, by email, and, if necessary, by phone. As will be described below, the sub-study will require in person assessments.
Screening: Individuals interested in learning more and potentially participating will log on to a secure website. Information about the study will be presented. If the individual is interested in joining the study he/she will complete questions relevant to our inclusion and exclusion criteria as well as sociodemographic questions (age, sex, and self-reported height and weight).
Main study: Based on the eligibility criteria for the larger study, participants will be directed to the study consent document, and if voluntary consent is given, they will be provided a link to the baseline battery. Participants agreeing to release their medical records will be directed to the PHI form on Docusign, where they will be asked to review and then sign and date the document.
Sub-study: If a participant meets the eligibility requirements for the sub-study, he or she will receive a message offering him/her the opportunity to participate. After reviewing the procedures and learning more about the procedures, a participant will have the option to join this study. If they decline participation, they will still have the opportunity to participate in the main study. If interested in the sub-study, they will be routed into a group for sub-study participants and will expect to hear from study staff to schedule an in person visit.
Clinic-visit (sub-study only): Participants interested in joining the sub-study will be contacted by a study staff member, who will guide them through the procedures and answer any questions that arise. Participants will then be scheduled for their first in-person clinic visit. The staff member will review the details of the visit, such as how to prepare and where to go.
The clinic visit assessment will consist of the following activities:

- Read and sign consent form

- Height/Weight measurement - to calculate BMI (confirm eligibility)
- Blood Pressure collection
- Blood Spot collection
- Waist to Hip Ratio collection
- Instruction on Morning and Nightly Diary procedures
- Orientation on Fitbit procedures

Baseline Questionnaire Battery (main study). This battery will be hosted by Qualtrics and all data will be secure. As part of this battery, participants will complete questionnaires to assess stress/distress symptoms as well the domain areas of mindfulness, health behaviors, physical health and work impact. Once completed, participants will be randomized to one of two groups: digital meditation group (Headspace) or waitlist control.
Baseline Questionnaire (Sub-study): The baseline questionnaire will be sent out to participants shortly after their visit, to complete on their own. Domain areas will be similar to the main study, with the addition of several health and eating behavior components.
Fitbit device (sub-study only): During the week prior to randomization and week 7 of the intervention period, participants will be asked to wear a Fitbit watch 24-hours per day. The Fitbit watch tracks information such as physical activity, sleep, heart rate and movement. Participants are asked to wear it at all times possible, with the exception of charging, cleaning, and syncing their device.
Daily Diary (sub-study only): During the week prior to randomization and week 7 of the intervention period, particiapnts are asked to complete a nightly diary and morning diary for 7 consecutive days. These diaries ask questions related to daily behaviors, sleep, mood and other related topics (see attached file).
Randomization (main-study)
Upon completing the baseline questionnaire, participants enrolled in the main study will be randomomized to the following conditions:
Digital Meditation Condition (Headspace): The intervention period will last for a total of 8-weeks (56 ± 2 days). Subjects in the Headspace group will be given simple instruction on how they should begin the journey (i.e. begin with Basics 1 followed by the Stress Pack, one 10 minute session per day). Subjects in the waitlist control group will not be given access to Headspace and will be instructed to refrain from any mindfulness or meditation. Active daily usage data in the Headspace group will be captured anonymously via the Headspace app and provided to UCSF as a part of the final analysis. App usage (engagement) will be tracked weekly for each participant.
Waitlist Condition: Participants in this condition will not receive any further study contact during the study window, with the exception of email links to questionnaire batteries at week 4 (midpoint assessment), week 8 (post-intervention), and 4-month follow-up. Once the 4-month follow up is completed or that time has lapsed, the study participant will be provided access to Headspace for the same duration (12 months) as is the case for those randomized to the digital meditation group.

Randomization (sub-study):

Upon completing the baseline questionnaire battery and 7 days of morning and nightly diaries and Fitbit measurement, participants will be randomized to one of the following study conditions:

Digital Meditation Condition (Headspace): The intervention period will last for a total of 8-weeks (56 ± 2 days). Subjects in the Headspace group will be given simple instruction on how they should begin the journey (i.e. begin with Basics 1 followed by the Stress Pack, one 10 minute session per day). Subjects in the waitlist control group will not be given access to Headspace and will be instructed to refrain from any mindfulness or meditation. Active daily usage data in the Headspace group will be captured anonymously via the Headspace app and provided to UCSF as a part of the final analysis. App usage (engagement) will be tracked weekly for each participant.

Healthy Eating condition: Those assigned to this condition will participate in a healthy eating program, designed to identify and assist problem eating behaviors, such as stress-related eating, which may contribute to metabolic problems. The program will include a 30- to 60-minute in-person counseling session at the beginning of the 8-week period along with 3 follow up booster phone calls and weekly text messages throughout the program. Following the counseling session, subjects will be given access to 2 online audio exercises to use when experiencing cravings and/or stress prior to eating/meal time. They will be required to access the audio tools at least once per week. One audio exercise uses mindful eating techniques to help participants make healthy eating choices, and the other audio exercise helps participants manage cravings for sweets and other tempting foods. Once the 4-month follow up is completed or that time has lapsed, the study participant will be provided access to Headspace for the same duration (12 months) as is the case for those randomized to the digital meditation group.

Counseling sessions: Once a participant is randomized into a condition that includes a healthy eating program, they will be contacted by phone, informed of their group randomization, offered a brief overview of the program, and coordinate a time and date to meet for an in-person 30- to 60-minute counseling session. In person counseling sessions will be conducted by a trained counselor (see script attached). During the counseling session, topics that will be covered include: a) psychoeducation regarding aspects of eating behaviors that contribute to metabolic dysfunction (e.g., cravings for sweets, stress-related eating, mindless eating), (b) identification of subject's triggers for overeating, cravings, and non-

homeostatic eating behaviors (d) goal-setting to reduce problematic eating behavior, and (c) introduction to mindful eating and cravings management exercises.

Healthy Eating and Digital Meditation Condition: This condition will last for a total of 8-weeks (56 ± 2 days). Subjects in this group will be asked to download and use the Headspace app for 10 minutes per day. Subjects in the Headspace group will be given simple instruction on how they should begin the journey (i.e. begin with Basics 1 followed by the Stress Pack, one 10 minute session per day). Active daily usage data in the Headspace group will be captured anonymously via the Headspace app and provided to UCSF as a part of the final analysis. App usage (engagement) will be tracked weekly for each participant. Additionally, participants will participate in a healthy eating program, designed to identify and assist problem eating behaviors, such as stress-related eating, which may contribute to metabolic problems. The program will include a 30- to 60-minute in-person counseling session (for more information, see below; these sessions will be audio recorded for treatment fidelity and research purposes) at the beginning of the 8-week period along with 3 follow up booster phone calls and weekly messages throughout the program. Following the counseling session, subjects will be given access to 2 online audio exercises to use when experiencing cravings and/or stress prior to eating/meal time. They will be required to access the audio tools at least once per week. One audio exercise uses mindful eating techniques to help participants make healthy eating choices, and the other audio exercise helps participants manage cravings for sweets.

Waitlist Condition: Participants in this condition will not receive any further study contact during the study window, with the exception of email links to questionnaire batteries at week 4 (midpoint assessment), week 8 (post-intervention), and 4-month follow-up. Once the 4-month follow up is completed or that time has lapsed, the study participant will be provided access to Headspace for the same duration (12 months) as is the case for those randomized to the digital meditation group.

Midpoint Questionnaire Battery (week 4). This battery will be hosted by Qualtrics and all data will be secure. An email link to this battery will be sent at week 4 for all study participants. This battery will be an abbreviated version of questionnaires completed at baseline, including stress/distress symptoms as well the domain areas of mindfulness, work impact, and for the sub-study, will include the domain of eating behaviors.

Post-intervention Questionnaire Battery (week 8). This battery will be hosted by Qualtrics and all data will be secure. This battery will serve as the post-intervention assessment and will be comprised of the same questionnaires included at the baseline assessment. Additionally, participants will be asked how often they have participated in a sitting meditation in the past 8 weeks. This battery will be made available to all participants via an email link.

Follow-up Questionnaire Battery (4-month follow-up (i.e., 2-months from end of intervention)). Participant s will receive a brief questionnaire battery similar to the midpoint assessment including measures of stress /distress symptoms as well the domain areas of mindfulness and work impact and health care utilization, and for the sub-study, will include the domain of eating behaviors.

Follow-up Questionnaire Battery (1-year follow-up (i.e., 10-months from end of intervention)). Participants who were initially randomized to the digital meditation condition will complete a brief questionnaire similar to the 4-month follow-up to assess the durability of the intervention, assess domains of distress, mindfulness, and work impact as well as health care utilization. The sub-study participants will also answer questions related to eating behaviors, similar to the baseline assessment.

Medical and employment record data: We are interested in whether meditation practice may affect health care utilization, job performance, and attendance. Participants will be option of making their electronic medical record and employee data available for use in this research study. All data will be collected and stored in a secured fashion, and like all the data in this study, will be deidentified. Participants can decline this request of access and continue to participate in this study.

Re-engagement protocol: As with any digital intervention study, attrition is common. In an effort to minimize attrition in this study, we have developed a re-engagement protocol. We will send an engagement text message to each participant starting about one week into their participation and for each subsequent week, for 7 weeks. The text message content for the intervention group will consist of gentle reminders to continue to meditate, along with tips to stay active and engaged in the study. For the waitlist control group, these weekly messages will consist of encouragements and brief reminders of participation requirements (see attached doc for specific text). If a participant does not engage after about 2 weeks, then he/she will receive a phone call from our research staff. For those participants who do not answer the phone, the study staff will leave a voicemail and a subsequent text message asking to call them back, and follow up again the next day with another text message. They only hear back from the staff in 2 weeks if they continue to refrain from the meditation app.

Sub-study: Participants will follow the same re-engagement protocol listed above, with minor modifications for the healthy eating groups.

Those in the healthy eating groups will participate in 3 separate booster phone calls (5-10 minutes) to check-in regarding progress and barriers towards healthy eating goals, and feasibility/acceptability of the brief eating intervention. Participants will receive automated text message pings approximately 3 times per week. The pings will provide reminders about mindful eating (including a link to audio sessions on cravings and mindful eating). The pings will occur during identified times of "high vulnerability" from the initial counseling session.

7.10 STANDARD CLINICAL PRACTICE: To what extent, if any, do the planned research procedures differ from the care that people would otherwise receive at this institution or the study site if not being done locally:

The standard care that participants receive will not change.

7.11 INSTRUMENTS: List all questionnaires, surveys, interview, or focus group guides that will be used for this study:

If the instruments are not complete or not available because they will be developed as part of this study, describe the basic content or include an outline and submit the final versions to the IRB with a modification for approval prior to use.

Eligibility Screening.

Basic Demographics: Participants will confirm their employment as UCSF employees, report their age, gender, assigned sex at birth, and height and weight.

Inclusion and exclusion criteria: Participants will be asked questions relating to our inclusion and exclusion criteria to ensure that they are eligible for the study.

Perceived Stress: The Perceived Stress Scale (PSS) is a gold standard measure for stress perceptions, including ratings of feeling overwhelmed, out of control, and stressed, and has been extensively validated (Cohen et al, 1988). (10 items).

Baseline Questionnaire Battery:

Socio-Demographics: household income, level of education, race/ethnicity, job position, UCSF campus location, as well as zip code and phone number for communication purposes. Participants will be given the option to opt-out of text messages.

Domain: Distress

Perceived Stress: The Perceived Stress Scale (PSS) is a gold standard measure for stress perceptions, including ratings of feeling overwhelmed, out of control, and stressed, and has been extensively validated (Cohen et al, 1988). (10 items).

Depressive symptoms: The Patient Health Questionnaire-9 (PHQ-9) is a nine-question multipurpose questionnaire to assess symptoms of depression and distress (Kroenke, Spitzer & Williams, 2001) (9 items)

Anxiety: Symptoms of anxiety will be assessed using the Generalized Anxiety Disorder-7 (Spitzer, Kroenke, Williams, & Löwe, 2006), which is a self-reported questionnaire and is routinely used in research (7 items).

Affect: Positive and negative affect will be measured by an 18-item self-report scale (Usala & Hertzog, 1989) that administers an adjective rating scale instrument measuring multiple affective states. Subjects are asked to rate the extent to which each adjective reflected their current mood on a 5-point Likert Scale.

Adverse Childhood Experiences: History of trauma will be evaluated using the Adverse Childhood Experiences Questionnaire, a 10-item survey asking participants to report whether or not they have experienced exposure to abuse or household dysfunction during the first 18 years of life (Felitti et al., 1998).

Domain: Non-homeostatic eating behaviors

Reward-Based Eating: The Reward-based Eating Drive (RED-9) scale is a 9-item self-report measure of reward-driven eating and captures a lack of satiety, preoccupation with eating, and loss of control over eating (Epel et al., 2014) (9 items).

USDA 1-item Food Insecurity Worry item: This is a 1-item measure that assesses the extent to which an individual experience worries over running out of food (1 item).

U.S. Household Food Security Module: The six-item short form of the survey module and the associated Six-Item Food Security Scale were developed by researchers at the National Center for Health Statistics in collaboration with Abt Associates Inc. and documented in "The effectiveness of a short form of the household food security scale," by S.J. Blumberg, K. Bialostosky, W.L. Hamilton, and R.R. Briefel (published by the *American Journal of Public Health*, vol. 89, pp. 1231-34, 1999). ERS conducted additional assessment of classification sensitivity, specificity, and bias relative to the 18-item scale.

The Sub-study will include the additional measures related to Eating:

Stress eating question (Epel, et al., 2004).We will assess *stress eating tendency* using the following 2 items: 1. How do you tend to eat on days when you feel moderately stressed? 2. How do you tend to eat on days when you feel extremely stressed? Item response choices range from *much less than usual* to *muc h more than usual*.

Questionnaire on Eating and Weight Patterns –5 (QEWP-5). The QEWP-5 (Yanovski, Marcus, Wadden, & Walsh, 2015) is a 24-item questionnaire that assesses frequency of reported binge eating. The QEWP-5 been adapted to capture loss of control (LOC) eating as well as binge episodes. This questionnaire is a screening tool designed to identify adults with possible DSM-5 (American Psychiatric Association, 2013) bulimia nervosa and binge eating disorder.

Palatable Eating Motives: Coping Subscale (PEMS)(Burgess, Turan, Lokken, Morse, & Boggiano, 2014). The PEMS Coping subscale is comprised of 4 Likert-like five-choice frequency response items that probe various motives for ‵‵eating tasty food and drinks.′′ The instructions include a list of examples of these kinds of foods and sugary drinks. The list of tasty foods was adopted from the Yale Food Addiction Scale (G earhardt, Corbin, & Brownell, 2009) with slight modifications. The Coping motives subscale measures intentionally using palatable food to cope with negative feelings (e.g., to forget about or help with worry, depression, nervousness, a bad mood, or problems). Item response choices range from *almost never /never* to *almost always/always.* The trait form for this subscale has been validated using ecological momentary assessment, and both versions are related equally to BMI and changes in emotional eating correlate with changes in BMI over time (Boggiano, Wenger, Turan, Tatum, Morgan, et al., 2015; Boggiano, Wenger, Turan, Tatum, Sylvester, et al., 2015).

Trait Food Craving Questionnaire, Reduced (FCQ-T-r)(Meule, Hermann, & Kubler, 2014). The Trait Food Craving Questionnaire, reduced, is a 15-item measure of behavioral, cognitive, and physical aspects of cravings for different types of food. Item response choices range from 0 (*never or n/a*) to 5 (*always*), and the total score is an average of the 15 items. High scores predict how much people crave and eat densely caloric snacks in daily life, and are associated with self-reported failures in dieting (Meule, Lutz, Vogele, & Kubler, 2012; Richard, Meule, Reichenberger, & Blechert, 2017)

Using Food to Cope: this a 2-item questionnaire asking respondents to indicate how they "usually experience a stressful event." Questions include: "I eat more of my favorite foods to make myself feel better" and I eat more than I usually do." Item response choices range from 1 (*a lot)* to 4 *(not at all)*. The responses to the items are reverse coded and summed so that higher scores indicate a greater use of food to cope with stress (Tsenkova, Boylan, & Ryff, 2013).

The Reward Based Eating Drive Scale (RED-13) (Mason, et al., 2017): The RED-13 is a 13-item self-report measure that broadly captures the spectrum of reward-related eating (RRE) and may be a useful tool for identifying individuals at risk for overweight or obesity. The RED-13 is designed to capture three dimensions of the RRE construct: lack of control over eating, lack of satiety, and preoccupation with food. RED-13 was also related to self-reported diagnosis of type 2 diabetes as well as cravings for sweet and savory foods (Mason, et al., 2017). Item response choices range from 0 (*strongly disagree)* to 4 (*strongly agree).*

Loss of Control over Eating Scale (LOCES) (Latner et al., 2014): The LOCES scale is a multidimensional scale designed to assess LOC eating in both clinical and nonclinical populations (Latner et al. 2014). Instructions include to ask participants to indicate how often during the past 28 days they have had the following experiences while eating. Each of the 7 questions has a 5- point scale from 1 (*Never)*, 2 (*Rarely),* 3 *(Occasionally),* 4 *(Often),* to 5 *(Always)*

Food Acceptance and Awareness Questionnaire (FAAQ) (Juarascio, Forman, Timko, Butryn, & Goodwin, 2011): The FAAQ was designed to measure the acceptance or ability to regulate eating despite urges and cravings and the desire to control these thoughts. The survey is comprised of 10 items and can be

answered using a 7-point likert rating scale ranging from 1 (*never true)* to 7 (*always true)*. Higher scores indicate greater acceptance of motivations to eat (Juarascio et al., 2011).

Food Frequency Questionnaire: (FFQ; modified from the Multi-ethnic Study of Atherosclerosis) (**Bild et al., 2002**), a 16-item self-report measure that assesses how frequently an individual eats foods from the following 16 food categories: processed meats, refined grains, whole grains, fruits, vegetables, white potatoes, fried foods, red meat, high-fat dairy, low-fat dairy, nuts/seeds/peanut butter, fish/seafood (not fried), sodas (non-diet), coffee, sweets, and alcohol. Items for each food group are answered on a Likert scale from 1 (rarely or never) to 9 (two times or more per day) in response to the question: *How often do you eat this type of food?* Individual scores for each food item are calculated as the frequency, ranging from 1 to 9.

Domain: Mindfulness

Mindfulness: This will be measured using the Mindful Attention Awareness Scale (MAAS) (Brown & Ryan, 2003), which assesses the tendency to be mindful day to day (15 items).

Mindwandering probes: We created for this study, a two item measure that assesses present mindwandering and the affective content of this mindwandering (attached).

Domain: Health Behaviors

Sleep Quality: overall sleep quality will be assessed using items from the Pittsburgh Sleep Quality Index (Buysse et al., 1989) to assess sleep onset latency, efficiency, duration, and subjective quality (5 items)

Physical activity: The Stanford Leisure-Time Activity (L-Cat) measures usual physical activity habits. We will use a 1-item measure to assess leisure physical activity (Kiernan et al., 2013) (1 item).

Domain: Physical Health

Self-reported health: This will be measured using the 12-item Short Form Survey (SF-12), which includes items related to mental and physical well-being and is consistently used in health research (12 items)

Stress-related somatic complaints: This measure was created for this study (attached) and asks participants to report on the extent they experience the following symptoms: body/joint pain, headaches, stomachaches, nausea, and cold/flu symptoms (5 items).

Checklist of medical and medication history: participants will complete a short check list on common medical conditions and medications they may or may not endorse.

Domain: Work

Work Engagement: Work engagement will be assessed using the 9 item self-report Utrech Work Engagement Scale (Schaufeli & Bakker 2003) which uses three factors to determine the level of work engagement: vigor, dedication and absorption.

Days of Work Missed: A two item self-report scale will measure the number of days participants have missed work due to either illness or non-illness related reasons.

Burnout: The Bergen Burnout Inventory is a 9-item measure that assesses three components associated with burnout, including exhaustion, cynicism at work, and sense of inadequacy at work (9 items).

Job Strain: The effort-reward imbalance scale will be used to assess job strain. It contains several subscales that measure effort, reward, and overcommitment and is used routinely in both research and occupational settings (Siegrist et al., 2004) (22 items).

Midpoint Questionnaire (week 4):

Mindfulness: This will be measured using the Mindful Attention Awareness Scale (MAAS) (Brown & Ryan, 2003), which assesses the tendency to be mindful day to day (15 items).

Perceived Stress: The Perceived Stress Scale (PSS) is a gold standard measure for stress perceptions, including ratings of feeling overwhelmed, out of control, and stressed, and has been extensively validated (Cohen et al, 1988). (10 items).

Job Strain: The effort-reward imbalance scale will be used to assess job strain. It contains several subscales that measure effort, reward, and overcommitment and is used routinely in both research and occupational settings (Siegrist et al., 2004) (22 items).

Work Engagement: Work engagement will be assessed using the 9 item self-report Utrech Work Engagement Scale (Schaufeli & Bakker 2003) which uses three factors to determine the level of work engagement: vigor, dedication and absorption.

The sub-study participants will also answer the following:
Palatable Eating Motives: Coping Subscale (PEMS) *(Burgess, Turan, Lokken, Morse, & Boggiano, 2014).* The PEMS Coping subscale is comprised of 4 Likert-like five-choice frequency response items that probe various motives for ‟eating tasty food and drinks.'' The instructions include a list of examples of these kinds of foods and sugary drinks. The list of tasty foods was adopted from the Yale Food Addiction Scale (Gearhardt, Corbin, & Brownell, 2009) with slight modifications. The Coping motives subscale measures intentionally using palatable food to cope with negative feelings (e.g., to forget about or help with worry, depression, nervousness, a bad mood, or problems). Item response choices range from *almost never /never* to *almost always/always.* The trait form for this subscale has been validated using ecological momentary assessment, and both versions are related equally to BMI and changes in emotional eating correlate with changes in BMI over time (Boggiano, Wenger, Turan, Tatum, Morgan, et al., 2015; Boggiano, Wenger, Turan, Tatum, Sylvester, et al., 2015).

Post intervention Questionnaire (week 8):

Participants will complete all the same questionnaire included in the baseline packet, with the exception of the medical and medication checklist and Adverse Childhood Events questionnaire. They will be asked about the frequency of their sitting meditation practice in the past 8 weeks.

Follow-up Questionnaire (2-months post intervention):

Mindfulness: This will be measured using the Mindful Attention Awareness Scale (MAAS) (Brown & Ryan, 2003), which assesses the tendency to be mindful day to day (15 items).

Perceived Stress: The Perceived Stress Scale (PSS) is a gold standard measure for stress perceptions, including ratings of feeling overwhelmed, out of control, and stressed, and has been extensively validated (Cohen et al, 1988). (10 items).

Job Strain: The effort-reward imbalance scale will be used to assess job strain. It contains several subscales that measure effort, reward, and overcommitment and is used routinely in both research and occupational settings (Siegrist et al., 2004) (22 items).

Work Engagement: Work engagement will be assessed using the 9 item self-report Utrech Work Engagement Scale (Schaufeli & Bakker 2003) which uses three factors to determine the level of work engagement: vigor, dedication and absorption.

The sub-study participants will also answer the following:
Palatable Eating Motives: Coping Subscale (PEMS) *(Burgess, Turan, Lokken, Morse, & Boggiano, 2014).* The PEMS Coping subscale is comprised of 4 Likert-like five-choice frequency response items that probe various motives for ‟eating tasty food and drinks.'' The instructions include a list of examples of these kinds of foods and sugary drinks. The list of tasty foods was adopted from the Yale Food Addiction Scale (Gearhardt, Corbin, & Brownell, 2009) with slight modifications. The Coping motives subscale measures intentionally using palatable food to cope with negative feelings (e.g., to forget about or help with worry, depression, nervousness, a bad mood, or problems). Item response choices range from *almost never /never* to *almost always/always.* The trait form for this subscale has been validated using ecological momentary assessment, and both versions are related equally to BMI and changes in emotional eating correlate with changes in BMI over time (Boggiano, Wenger, Turan, Tatum, Morgan, et al., 2015; Boggiano, Wenger, Turan, Tatum, Sylvester, et al., 2015).

Follow-up Questionniare (10-months post intervention; digital meditation condition only):

Mindfulness: This will be measured using the Mindful Attention Awareness Scale (MAAS) (Brown & Ryan, 2003), which assesses the tendency to be mindful day to day (15 items).

Perceived Stress: The Perceived Stress Scale (PSS) is a gold standard measure for stress perceptions, including ratings of feeling overwhelmed, out of control, and stressed, and has been extensively validated (Cohen et al, 1988). (10 items).

Job Strain: The effort-reward imbalance scale will be used to assess job strain. It contains several subscales that measure effort, reward, and overcommitment and is used routinely in both research and occupational settings (Siegrist et al., 2004) (22 items).

Work Engagement: Work engagement will be assessed using the 9 item self-report Utrech Work Engagement Scale (Schaufeli & Bakker 2003) which uses three factors to determine the level of work engagement: vigor, dedication and absorption.

Participants may also complete the following:
U.S. Household Food Security Module: The six-item short form of the survey module and the associated Six-Item Food Security Scale were developed by researchers at the National Center for Health Statistics in collaboration with Abt Associates Inc. and documented in "The effectiveness of a short form of the

household food security scale," by S.J. Blumberg, K. Bialostosky, W.L. Hamilton, and R.R. Briefel (published by the *American Journal of Public Health*, vol. 89, pp. 1231-34, 1999). ERS conducted additional assessment of classification sensitivity, specificity, and bias relative to the 18-item scale.

The sub-study participants will also answer the following:
Palatable Eating Motives: Coping Subscale (PEMS) (Burgess, Turan, Lokken, Morse, & Boggiano, 2014). The PEMS Coping subscale is comprised of 4 Likert-like five-choice frequency response items that probe various motives for "eating tasty food and drinks." The instructions include a list of examples of these kinds of foods and sugary drinks. The list of tasty foods was adopted from the Yale Food Addiction Scale (Gearhardt, Corbin, & Brownell, 2009) with slight modifications. The Coping motives subscale measures intentionally using palatable food to cope with negative feelings (e.g., to forget about or help with worry, depression, nervousness, a bad mood, or problems). Item response choices range from *almost never /never* to *almost always/always.* The trait form for this subscale has been validated using ecological momentary assessment, and both versions are related equally to BMI and changes in emotional eating correlate with changes in BMI over time (Boggiano, Wenger, Turan, Tatum, Morgan, et al., 2015; Boggiano, Wenger, Turan, Tatum, Sylvester, et al., 2015).

Attach any unpublished instruments in the 'Other Study Documents' section of the Initial Review Submission Packet form after completing the study application. Published instruments should NOT be attached.

7.12 * BIOSPECIMEN COLLECTION: Are you drawing any blood or collecting other biosamples (e.g. tissue, buccal swabs, urine, saliva, hair, etc.) for analysis under this protocol and/or storage for future research: (REQUIRED)

○ Yes ◉ No

7.13 STATISTICAL METHODS: Briefly summarize the methods and types of analyses that will be performed:

We will employ chi-square and *t*-tests to examine group difference in baseline in demographics and measures obtained by our baseline questionnaire. Intervention effects on each outcome were tested using linear mixed models with Time (t1 and t2) was included as a within-person factor and intervention group as a between-person factor. We will analyze the data using an intent-to-treat framework, and carry forward latest data point in our final analysis. Alpha's were set at .05 for each outcome. We used eta-squared as our effect size index.

To investigate dose response effects, we will examine whether number of mediation sessions moderates the interventon effects among those in the digital meditation group. In this regard, we will test a 2-way interaction (number of weekly meditation*time). All analyses will be conducted in SPSS Version 24 and STATA 13.1.

7.14 REFERENCES: List only the 5-10 most relevant references (a separate bibliography can be attached for reference purposes if this study involves novel approaches, agents, or an emerging technology that the IRB may not be familiar with):

Ganster, D. C., & Rosen, C. C. (2013). Work stress and employee health. *Journal of Management*, *39*(5), 1085–1122. https://doi.org/10.1177/0149206313475815

Goh, J., Pfeffer, J., & Zenios, S. A. (2016). The Relationship Between Workplace Stressors and Mortality and Health Costs in the United States. *Management Science*, *62*(2), 608–628. https://doi.org/10.1287/mnsc.2014.2115

Karasek, R. A. (1979). Job Demands, Job Decision Latitude, and Mental Strain: Implications for Job Redesign. *Administrative Science Quarterly*, *24*(2), 285. https://doi.org/10.2307/2392498

Landsbergis, P. A., Dobson, M., Koutsouras, G., & Schnall, P. (2013). Job strain and ambulatory blood pressure: A meta-analysis and systematic review. *American Journal of Public Health*, *103*(3), e61-71. https://doi.org/10.2105/AJPH.2012.301153

Landsbergis, P. A., Schnall, P. L., Warren, K., Pickering, T. G., Schwartz, J. E., & Paul Landsbergis, by A. (1994). Association between ambulatory blood pressure and alternative formulations of job strain. *So urce: Scandinavian Journal of Work, Environment & Health Scandinavian Journal of Work ScandJ Work Environ Health*, (5).

Ly, K. H., Trüschel, A., Jarl, L., Magnusson, S., Windahl, T., Johansson, R., … Andersson, G. (2014). Behavioural activation versus mindfulness-based guided self-help treatment administered through a smartphone application: A randomised controlled trial. *BMJ Open*, *4*(1), e003440. https://doi.org/10.1136/bmjopen-2013-003440

Mani, M., Kavanagh, D. J., Hides, L., & Stoyanov, S. R. (2015). Review and evaluation of mindfulness-based iPhone apps. *JMIR mHealth and uHealth*, *3*(3), e82. https://doi.org/10.2196/mhealth.4328

Roeser, R. W., Schonert-Reichl, K. A., Jha, A., Cullen, M., Wallace, L., Wilensky, R., … Harrison, J. (2013). Mindfulness training and reductions in teacher stress and burnout: Results from two randomized, waitlist-control field trials. *Journal of Educational Psychology*, *105*(3), 787–804. https://doi.org/10.1037/a0032093

Siegrist, J., Starke, D., Chandola, T., Godin, I., Marmot, M., Niedhammer, I., & Peter, R. (2004). The measurement of effort-reward imbalance at work: European comparisons. *Social Science and Medicine*, *58*(8), 1483–1499. https://doi.org/10.1016/S0277-9536(03)00351-4

The Sainsbury Centre for Mental Health. (2007). *Mental Health at Work: Developing the business case* (Vol. 8).

Virgili, M. (2015). Mindfulness-based interventions reduce psychological distress in working adults: A meta-analysis of intervention studies. *Mindfulness*, *6*(2), 326–337. https://doi.org/10.1007/s12671-013-0264-0

Wolever, R. Q., Bobinet, K. J., McCabe, K., Mackenzie, E. R., Fekete, E., Kusnick, C. A., & Baime, M. (2012). Effective and Viable Mind-Body Stress Reductin in the Workplace: A Randomized Controlled Trial. *Journal of Occupational Health Psychology*, *17*(2), 246–58. https://doi.org/10.1037/a0027278

8.0 Drugs and Devices

8.1 * DRUGS AND/OR BIOLOGICS: Are you STUDYING any drugs and/or biologics that are either approved or unapproved: (REQUIRED)

○ Yes ◉ No

8.3 * MEDICAL DEVICES: Are you STUDYING any medical devices, in vitro diagnostics, or assays that are either approved or unapproved:(REQUIRED)

○ Yes ◉ No

9.0 Sample Size and Eligibility Criteria

9.1 ENROLLMENT TARGET: How many people will you enroll:

10000

If there are multiple participant groups, indicate how many people will be in each group:

We will screen a high number of potential participants to carry out this study. In this regard, we will screen as many as 10000 individuals and thus as many as 10000 individuals will complete a consent form to be screened. Of those who complete the screening, we anticipate that we will consent into the study 2000 participants. We will randomize 1000 participants to our digital mediation condition (Headspace) and 1000 to the waitlist control condition.

9.3 SAMPLE SIZE JUSTIFICATION: Explain how and why the number of people was chosen. For multi-site studies, this is referring to the number that will be enrolled across all sites:

We are interested in testing a digital intervention with limited contact by study investigators (i.e., no in person contact). As such, participant attrition is to be expected. Our prior study (Bostock et al., under revision) detected effects in a sample of < 250 participants. We expect that our sample size will be well powered to detect improvements in our self-report measures in response to our treatment intervention.

9.4 * PARTICIPANT AGE RANGE: Eligible age ranges: (REQUIRED)

- [ ] 0-6 years
- [ ] 7-12 years
- [ ] 13-17 years
- [x] 18-64 years
- [x] 65+

9.5 * STUDY POPULATIONS: Data will be collected from or about the following types of people (check all that apply): (REQUIRED)

- [ ] Inpatients
- [ ] Outpatients
- [ ] Family members or caregivers
- [ ] Providers
- [ ] People who have a condition but who are not being seen as patients
- [ ] Healthy volunteers
- [ ] Students
- [x] Staff of UCSF or affiliated institutions
- [ ] None of the above

9.6 * SPECIAL SUBJECT GROUPS: Check the populations that may be enrolled: (REQUIRED)

- [ ] Children / Minors
- [ ] Adult subjects unable to consent for themselves
- [ ] Adult subjects unable to consent for themselves (emergency setting)
- [ ] Subjects with diminished capacity to consent
- [ ] Subjects unable to read, speak or understand English
- [ ] Pregnant women
- [ ] Fetuses
- [ ] Neonates
- [ ] Prisoners
- [ ] Economically or educationally disadvantaged persons

☑ None of the above

9.7 INCLUSION CRITERIA: Briefly describe the population(s) that will be involved in this study. Include anyone that data will be collected from or about (e.g. patients, healthy controls, caregivers, providers, administrators, students, parents, family members, etc.):

A subject will be eligible for inclusion in this study only if all of the following criteria apply:

- Have access to a smartphone or computer every day
- Fluent in English and has at least a third grade reading level
- Is a UCSF employee
- Perceived Stress Scale total of score of 15 or higher
- Consent: demonstrates understanding of the study and willingness to participate as evidenced by voluntary informed consent and has received a signed and dated copy of the informed consent form
- Is at least 18 years of age
- Expresses willingness to be randomly assigned into with the waitlist group or the intervention group

Substudy participants must report a BMI of 25 or above

9.8 EXCLUSION CRITERIA: List any exclusion criteria (e.g. reasons why someone would not be included in the study):

A subject will not be eligible for inclusion in this study if any of the following criteria apply:

- Experienced meditator and/or report current (three times per week or more- for 10 minutes at a time) sitting meditiation practice

9.9 * RESEARCH CONDUCTED ON PATIENT CARE WARDS: Do any study activities take place on any patient care units including inpatient wards, peri- or post-operative care units, operating rooms, or in the Emergency Department at UCSF Health medical facilities: (REQUIRED)

○ Yes ◉ No

9.11 * EMERGENCY DEPARTMENT: Does your protocol or study involve any of the following patient related activities in the emergency department (e.g. subject identification, recruitment, consent, blood draws, specimen retrieval, involvement of ED staff (nursing, tech, and/or physician), or any other ED based procedures): (REQUIRED)

○ Yes ◉ No

10.0 Recruitment and Consent

10.1 * COMPETITIVE ENROLLMENT: Is this a competitive enrollment clinical trial? By competitive enrollment, we mean that sites who do not enroll participants early may not get to participate at all: (REQUIRED)

○ Yes ◉ No

10.2 * SUBJECT IDENTIFICATION METHODS: What kinds of methods will be used to identify potential

participants for recruitment (check all that apply): (REQUIRED)

- [ ] Review of patients' conditions, history, test results, etc. (includes patients seen in clinic, scheduled for surgery, a procedure, imaging, or tests, or seen in the Emergency Department as well as searching through medical record data for possible cohort identification)
- [ ] Already approved recruitment registry
- [x] Re-contact of participants from the investigators' previous studies
- [x] Referrals from colleagues (attach the 'Dear Colleague' letter or other recruitment materials you will provide to colleagues)
- [ ] Referrals from the community / word of mouth
- [x] Advertisements (flyers, brochures, radio or t.v. ads, posting on clinical research sites or social media, presentation of the study at community events/media, etc.)
- [x] Online recruiting tool (describe below)
- [ ] CTSI Recruitment Services unit
- [ ] Posting on UCSF Clinical Trials, ClinicalTrials.gov or other publicly available clinical trial website
- [ ] Other method (describe below)

Attach your recruitment materials (e.g., flyers, ads, recruitment letter templates, email text, etc.) in the Other Study Documents section of the Initial Review Submission Packet Form.

* Provide details about the subject identification methods: **(REQUIRED)**

UCSF employees will be recruited through flyers, staff events, email, etc.

* Did all the participants of previous studies provide permission to be contacted for future studies: **(REQUIRED)**

(•) Yes ( ) No

10.4 DETERMINATION OF ELIGIBILITY: How, when, and by whom will eligibility for recruitment be determined:

Any UCSF employees will be eligible for recruitment. They will be targeted at UCSF campuses and utilizing university services such as flyers, advertisements, digital media, emails through listservs. We expect to reach as many as 10,000 individuals.

Those interested in participation will inquire on the website for more details, and their eligibility for the study will be determined by the screening questionnaire. If they are eligible, they will have the option to consent and will be invited into the study via an automated email.

10.5 * INITIATION OF CONTACT: Who initiates contact (check all that apply): (REQUIRED)

- [x] Investigators/study team
- [ ] UCSF recruitment unit (e.g. CTSI Consultation Services)
- [ ] Potential participant
- [ ] Other (explain below)

10.6 * HOW IS CONTACT INITIATED: (check all that apply): (REQUIRED)

- [x] In person
- [ ] Phone
- [x] Letter / email
- [x] Website or app
- [ ] Other (explain below)

Attach the recruitment letter or email template in the Other Study Documents section of the Initial Review Submission Packet Form.

Provide the URL for any website in Recruitment Plan section, or attach a mock-up of the website or the app screens in the Other Study Documents section of the Initial Review Submission Packet Form.

10.7 RECRUITMENT PLAN: Based on the checkboxes you chose above, please provide a narrative describing your recruitment plan. We want to know:

- **Who is conducting the search for potential participants, and how?**
- **How are potential subjects being approached for recruitment? By whom, and when?**

If there will be more than one participant group (e.g. patients, healthy controls, caregivers, family members, providers, etc.), provide details about the recruitment plans for each group. (Recommended length - 100-250 words)

The research staff, including a clinical research coordinator and research assistants, will recruit participants using several strategies, including:

- Flyers at each of the UCSF campuses, including in cafeterias and other areas of high traffic areas, such as UCSF Shuttles
- UCSF wide emails via announcements and staff listservs (email blasts)
- Digital advertisements posted on screens around campus and on social media
- Announcements about the study in lectures or other on-campus events

10.8 * CONSENT METHODS: How will permission to participate (i.e., informed consent) be obtained from each potential participant. If there will be multiple groups and different plans for consenting each, check all that apply. See the orange Help bubble to the right for more detailed guidance. Participants will (check all that apply): (REQUIRED)

- [x] Sign a consent form at the end of the consent discussion (signed consent)
- [x] Provide online 'eConsent' using an E-Signature system
- [x] Click through a link in a survey or email after reading about the study and then complete the study online (electronic consent)
- [ ] Be told about the study and be given a handout/information sheet and be asked if they agree to participate (verbal consent)
- [ ] Complete the study activities and turn in materials, as in the case of a completed survey that is placed in a drop box or mailed to the study team (implied consent)
- [ ] Not be able to provide consent and will have a family member consent for them, as in the case of a critically ill or unconscious patient (surrogate consent)
- [ ] Not be able to provide consent (emergency waiver of consent - allowed for minimal risk research or greater than minimal risk research with an approved community consultation plan)
- [ ] Not know about the study, as in the case of chart reviews or observations of public behavior (waiver of consent)
- [ ] Other method (describe below)

Attach your consent form, information sheet, or electronic consent text in the Informed Consent Documents section of the Initial Review Submission Packet Form.

10.9 * CONSENT PROCESS: Describe the process for obtaining informed consent, including details such as who will have the consent discussion and when participants will be asked to sign the consent form in relation to finding out about the study: (REQUIRED) We encourage researchers to review our guidance on obtaining and documenting informed consent.

- **If there are multiple groups being consented differently, provide details about the consent process for each group.**

- **If you are relying on verbal or implied consent, provide details about how that will happen.**
- **For studies using online recruitment and consent or consent via mail, provide details here.**

Potential participants will go to the website of our study and on the homepage, they can choose to "join the study." After completing the eligibility screening questionnaire, eligible participants will be routed to the online informed consent page, with the bill of rights and the complete IRB approved informed consent document. They will be provided detailed information about the study and will be instructed to read through and check the box confirming that they "have read, understood and agreed to the consent process." They will have the option to print or save the document as a pdf. They will also be provided with a study email address as a contact if they have specific questions. They will be informed of their voluntary participation and that none of the information collected will have any impact on their employment at UCSF.

Sub-study: Potential participants will read and sign a printed version of the informed consent at the end of the consent discussion at the first in-person assessment. The study staff will provide a signed copy to the participant if requested, and save a copy in a secure, locked location in the lab.

* It is important that the people obtaining consent are qualified to do so. Briefly describe the training and experience these individuals have in obtaining informed consent: **(REQUIRED)**

All Research Assistants will complete thorough training on consent process, including reviews of IRB protocol, undergo HIPAA certification, and are required to complete CITI training.

10.10 * CONSENT COMPREHENSION: Indicate how the study team will assess and enhance the subjects' understanding of study procedures, risks, and benefits prior to signing the consent form (check all that apply): (REQUIRED) Tip: Review the Consent Comprehension - Learning Notes in the Help bubble at the right for specific questions that can be asked to assess comprehension, consider using the UCSF Decision-Making Capacity Assesment Tool, and review our guidance on obtaining written or verbal informed consent for more detail on how to conduct the assessment.

- [x] The study team will engage the potential participant in a dialogue, using open-ended questions about the nature of the study or the experimental treatment, the risks and benefits of participating, and the voluntary nature of participation
- [x] Potential participants will be asked or shown a series of questions to assess their understanding of the study purpose, procedures, risks and benefits, as well as the voluntary nature of participation (especially appropriate when the consent process happens online or through a mobile health app)
- [ ] Other method (describe below):

Provide details of the other approaches that will be used, if using another method to assess comprehension:

10.11 * DECEPTION: Does this study rely on some deception or misinformation about what the researchers are observing to get valid data? (REQUIRED)

○ Yes ◉ No

10.13 * WAIVER OF DOCUMENTATION OF SIGNED CONSENT: Select the regulatory category under which the IRB may waive the requirement to obtain *signed* consent for this study:

○ The only record linking the subject and the research would be the consent document and the principal risk would be potential harm resulting from a breach of confidentiality. Each subject will be asked whether they want documentation linking them with the research. 46.117(c) (1)

◉ The research presents no more than minimal risk of harm to subjects and involves no procedures for which written consent is normally required outside of the research context. 46.117(c) (2)

10.14 TIME: What is the estimated time commitment for participants (per visit and in total):

The propsosed study is designed to be carried out digitally with limited contact with study staff. All participants will be asked to complete our questionnaire batteries at baseline, week 4, week 8 and + 4 months, and participants randomized to the digital meditation condition will be asked to complete + 1 year follow up questionnaire battery. The questionnaires included at the baseline and week 8 time points require 20 minutes to complete while the questionaires completed at the other time points are much shorter, requiring between 5 to 10 minutes each time. Additionally, participants randomized to the digital meditation condition will be asked to use the Headspace app each day (10 minutes daily) over the 8 week study.

Sub-study:
All participants in the sub-sample will be asked to complete questionnaire batteries at baseline, week 4, week 8 and + 4 months, and participants randomized to the digital meditation condition will be asked to complete + 1 year follow up questionnaire battery. The questionnaires included at the baseline and week 8 time points require about 30-35 minutes to complete while the questionnaires completed at the other time points are much shorter, requiring between 5 to 10 minutes each time.

In person assessments: The first in-person assessment will last no longer than 60 minutes, and the final assessment is estimated to require 30 minutes, not counting transportation to the meetings.

Daily diary: There will be fourteen total surveys, each lasting about 3-5 minutes.

Headspace meditations: The digital meditation condition will be asked to use the Headspace app each day (10 minutes daily) over the 8 week study.

Healthy eating program: Either one of the conditions including the healthy eating program will be involved in additional activities. First, a phone call to schedule the counseling session is expected to last about 5-10 minutes, while the counseling visit itself is estimated to last 30 to 60 minutes. Next, the 3 phone calls received throughout the duration of the intervention will last about 5-10 minutes each. The audio modules require about 5 minutes each, and participants are required to access at least one module at least once per week.

IMPORTANT TIP: Ensure this information is consistent with the information provided in the consent form.

10.15 ALTERNATIVES: Is there a standard of care (SOC) or usual care that would be offered to prospective participants at UCSF (or the study site) if they did not participate in this research study:

○ Yes ◉ No

10.16 OFF-STUDY TREATMENT: Is the study drug or treatment available off-study:

◉ Yes
○ No
○ Not applicable

10.17 OTHER ALTERNATIVES: Describe other alternatives to study participation, if any, that are available to prospective subjects:

There is no relevant alternative to study participation. If they choose not to participate, they will continue to receive all benefits of employment at the university.

11.0 Risks and Benefits

11.1 RESEARCH-RELATED RISKS: Check if your study involves any of these specific research-related risks to participants that may need to be disclosed in the consent form:

☑ Physical discomforts or pain
☐

Risks to employment, or social or legal standing

- [ ] Risk that the study team may observe possible evidence of child abuse, elder abuse, or a threat to self or others that they are required to report

* For any boxes checked above, describe how you will minimize these risks and discomforts, e.g., adding or increasing the frequency of monitoring, additional screening to identify and exclude people with diminished kidney or liver function, or modification of procedures such as changing imaging studies to avoid giving contrast agent to people who are more likely to suffer side effects from it, etc.: **(REQUIRED)**

Because questions about stress, job strain, and emotion are sensitive, participants will be told in the consent form that if they experience any discomfort or negative emotions when answering assessments or meditating, they can stop at any time. The online survey database (Qualtrics) allows subjects to pause and return to the questionnaire as they left off. They also have the option to skip any questions that they don't want to answer.

Because collecting blood pressure and conducting a finger prick to draw blood can be uncomfortable or painful experiences, participants are told during the consent process that they may take a pause or stop all together. If they feel any pain, they may let the research assistant know and the assessment can be discontinued. Moreover, the research assistants will be trained to minimize discomfort, and will offer ice to reduce any swelling if applicable.

11.2 * RISKS: Describe any anticipated risks and discomforts not listed above: (REQUIRED)

Potential risks and discomforts are minimal.

- Some of the surveys may produce some unpleasant feelings when talking about or reporting personal topics that may be sensitive. If this occurs, participants will be informed that they can skip any questions that make them uncomfortable. They have the option to take a break and return to them later are free to stop at any time.
- Confidentiality: Participation in any kind of research may involve a loss of privacy, but information will be handled as confidentially as possible. All research data generated from the proposed work will be kept in locked files, secure servers or encrypted laptops at UCSF by the principal investigator of the study, with access restricted to study staff.
- Although it is unlikely, participants may experience difficult emotions that may arise as they meditate. They are free to stop or take a break at any time.

Sub-study:

- Blood pressure assessments require a tight squeeze on your upper arm that can be unpleasant.

- Blood sampling will be carried out using a fingerstick, similar to what is used by patients with Type 2 diabetes, and there is a slight chance that the finger prick may result in swelling, bruising, and /or infection.

11.3

MINIMIZING RISKS: Describe the steps you have taken to minimize the risks/discomforts to subjects. Examples include:

- **designing the study to make use of procedures involving less risk when appropriate**
- **minimizing study procedures by taking advantage of clinical procedures conducted on the study participants**
- **mitigating risks by planning special monitoring or conducting supportive interventions for the study**
- **having a plan for evaluation and possible referral of subjects who report suicidal ideation**

Because questions about stress and emotion are sensitive, participants will be told in the consent form that if they experience any discomfort or negative emotions when answering assessments or meditating, they can stop at any time. The online survey database (Qualtrics) allows subjects to pause and return to the questionnaire as they left off. They also have the option to skip any questions that they don't want to answer.

There will be minimal risk of loss of privacy since participants are anonymous and **no identifying data will be collected except contact information.** Data will be stored in a secure place on encrypted files that only research staff will have access to. The potential harm from having identified responses released is low, except for possible feelings of embarassment. Additionally, we will store identifying information in a separate file from study data, linked by a study ID number.

Sub-study:
During the collection of blood pressure, participants are told that if they are uncomfortable, to please let the research assistant know and the blood pressure assessment can be discontinued.

In an effort to minimize discomfort, this study uses the smallest lancet possible; however, the finger prick can be slightly painful. The research staff has undergone training to minimize any discomfort. They will also provide ice to reduce any swelling if requested. Additionally, they may ask study staff to pause or discontinue the assessment altogether. To minimize risk of infection, both study staff and participant will wash their hands prior to finger prick, in addition to thoroughly sterilizing the finger pad using a sterile wipe prior to the finger prick.

11.5 * BENEFITS: (REQUIRED) Note: These are the benefits that the IRB will consider during their review. They are not necessarily appropriate to include in the consent form.

Possible immediate and/or direct benefits to participants and society at large (check all that apply):

- [x] Positive health outcome (e.g. improvement of condition, relief of pain, increased mobility, etc.)
- [ ] Closer follow-up than standard care may lead to improved outcomes or patient engagement
- [ ] Health and lifestyle changes may occur as a result of participation
- [ ] Knowledge may be gained about their health and health conditions
- [x] Feeling of contribution to knowledge in the health or social sciences field
- [x] The research presents a reasonable opportunity to further the understanding, prevention, or alleviation of a serious problem affecting the health or welfare of children
- [x] Other benefit (describe below)
- [ ] None

Briefly discuss the other possible benefits:

Participation in this study may result in reduced stress and improved psychological well-being.

Participation in the sub-study may lead to the above, as well as better eating behaviors.

11.6 RISK TO BENEFIT RATIO: Explain why the risks to subjects are reasonable in relation to anticipated benefits, if any, to the participant or society:

The potential risks are minimal and have a low probability of occurring. The anticipated benefits, both to the participant and to society, are greater in relation to risks. Participants may benefit from the use of the digital meditation application, potentially experiencing decreases in overall stress, increases in mindfulness and other related markers of wellbeing. All study participants will gain free access to the Headspace application for one year, which normally costs a monthly fee. The results of this study may also benefit the larger society by contributing scientitic knowledge about stress and the potential benefits of an app-based meditation program.

Sub-study:

Participants in the study may benefit by receiving a Fitbit Charge 3 Watch as part of the study protocol. The retail value of the watch is $130, and ownership may have positive benefits to the study participants.

11.7 * DATA AND SAFETY MONITORING: Do you have a Data and Safety Monitoring Plan (DSMP) for this study (A DSMP is required for Greater than Minimal Risk research): (Click the Help link for guidance on risk determination) (REQUIRED)

( ) Yes (•) No

This is not required for minimal risk research but the UCSF IRB strongly recommends one to ensure the data collected are adequate to meet the research aims:

12.0 Confidentiality, Privacy, and Data Security

12.1 PROTECTING PRIVACY: Indicate how subject privacy will be protected:

- [ ] Conduct conversations about the research in a private room
- [ ] Ask the subject how they wish to be communicated with – what phone numbers can be called, can messages be left, can they receive mail about the study at home, etc.
- [x] Take special measures to ensure that data collected about sensitive issues do not get added to their medical records or shared with others without the subject's permission
- [ ] Other methods (describe below)

12.2 SENSITIVE DATA: Do any of the instruments ask about illegal or stigmatized behavior:

○ Yes ◉ No

12.3 SIGNIFICANT CONSEQUENCES OF A LOSS OF PRIVACY OR CONFIDENTIALITY: Could a breach of privacy or confidentiality result in any significant consequences to participants, such as criminal or civil liability, loss of state or federal benefits, or be damaging to the participant's financial standing, employability, or reputation:

◉ Yes ○ No

Check all that apply:

- [x] Embarrassment
- [ ] Criminal or civil liability
- [ ] Loss of state or federal benefits
- [ ] Damaging to the participant's financial standing, employability, or reputation
- [ ] Potential risks to insurability (health, disability, or life insurance)

Describe the potential consequences:

There are minimal foreseeable risks to one's reputation or insurability from participating in this study. While we don't expect a loss of privacy, it could result in embarrassment or shame. Otherwise, there is low risk of any other serious consequences to participants.

12.4 EXTRA CONFIDENTIALITY MEASURES: Explain any extra steps that will be taken to assure confidentiality and protect identifiable information from improper use and disclosure, if any:

Data will be stored on secure university servers or encrypted hard drives. Additionally, we will store identifying information in a separate file from study data, linked by a study ID number. The key linking the subject's identity to their ID number will be kept in a confidential manner in a locked file cabinet at UCSF inside a locked office, or on a secure server (password-protected), with access only by the project investigators and the research staff.

12.5 * REPORTABILITY: Do you anticipate that this study may collect information that State or Federal law requires to be reported to other officials, such as elder abuse, child abuse, or threat to self or others: (REQUIRED)

◯ Yes ◉ No

12.6 CERTIFICATE OF CONFIDENTIALITY: Will this study obain a Certificate of Confidentiality:

◯ Yes ◉ No

12.7 SHARING OF RESEARCH RESULTS: Will there be any sharing of EXPERIMENTAL research test results with subjects or their care providers:

◯ Yes ◉ No

12.9 * HIPAA APPLICABILITY: Study data will be: (REQUIRED)

- [x] Derived from a medical record (e.g. APeX, OnCore, etc. Identify source below)
- [ ] Added to the hospital or clinical medical record
- [ ] Created or collected as part of health care
- [ ] Used to make health care decisions
- [x] Obtained from the subject, including interviews, questionnaires
- [ ] Obtained ONLY from a foreign country or countries
- [ ] Obtained ONLY from records open to the public
- [ ] Obtained from existing research records
- [ ] None of the above
- [ ] Derived from the Integrated Data Repository (IDR) or The Health Record Data Service (THREDS) at SFGH

In addition to signing a consent form, each subject will have to sign the UCSF Research Subject Authorization Form (HIPAA Form). Upload the HIPAA Authorization Form in the Other Study Documents section of the Initial Review Submission Packet Form. Failure to have patients sign the HIPAA Authorization is one of the most common findings from QIU Routine Site Visits. Please call the IRB office at 415-476-1814 if you have questions about HIPAA research requirements.

If derived from a medical record, identify source:

Apex

12.10 * IDENTIFIERS: Check all identifiers that will be collected and included in the research records, even temporarily: (REQUIRED)

◉ Yes ◯ No

* Check all the identifiers that may be included **(REQUIRED)**:

- [x] Names
- [x] Dates
- [x] Postal addresses
- [x] Phone numbers
- [ ] Fax numbers
- [x] Email addresses
- [ ] Social Security Numbers*
- [x] Medical record numbers
- [ ] Health plan numbers

- [ ] Account numbers
- [ ] License or certificate numbers
- [ ] Vehicle ID numbers
- [ ] Device identifiers or serial numbers
- [ ] Web URLs
- [x] IP address numbers
- [ ] Biometric identifiers
- [ ] Facial photos or other identifiable images
- [ ] Any other unique identifier
- [ ] None

* Could study records include *ANY* photos or images (even 'unidentifiable' ones): **(REQUIRED)**

( ) Yes (•) No

12.14 * HIPAA - PERMISSION TO ACCESS SENSITIVE DATA: Does the research require access to any of the following types of health information from the medical record: (check all that apply) (REQUIRED)

- [x] Drug or alcohol abuse, diagnosis or treatment
- [ ] HIV/AIDS testing information
- [ ] Genetic testing information
- [x] Mental health diagnosis or treatment
- [x] None of the above

Important note: Ensure that participants initial the corresponding line(s) in Section C of the HIPAA authorization form during the consent process.

12.18 * DATA COLLECTION AND STORAGE: (check all that apply): (REQUIRED)

Collection methods:

- [ ] Electronic case report form systems (eCRFs), such as OnCore or sponsor-provided clinical trial management portal
- [ ] UCSF ITS approved Web-based online survey tools: Qualtrics or RedCap
- [x] Other web-based online surveys or computer-assisted interview tool
- [x] Mobile applications (mobile or tablet-based)
- [ ] Text Messaging
- [ ] Wearable devices
- [ ] Audio/video recordings
- [ ] Photographs
- [ ] Paper-based (surveys, logs, diaries, etc.)
- [ ] Other:

* For each app and device, please provide: **(REQUIRED)**

- the name of the mobile application or wearable device
- name of the manufacturer / application owner
- the FDA status (required for mobile health applications and mobile health devices)

Name: Headspace
Manufacturer: Headspace Meditation Ltd

* Data will be collected/stored in systems owned by (check all that apply): **(REQUIRED)**

- [ ] Study sponsor
- [x] UCSF data center (including OnCore, RedCap, Qualtrics, and MyResearch)
- [ ] UCSF encrypted server, workstation, or laptop residing outside of UCSF data center
- [ ] Personal devices, such as laptops or tablets that are not owned or managed by UCSF
- [ ] SF VAMC
- [ ] Zuckerberg San Francisco General Hospital
- [ ] Benioff Children's Hospital Oakland
- [ ] Langley Porter Psychiatric Institution
- [ ] Other UCSF affiliate clinic or location (specify below)
- [ ] Cloud vendor such as Amazon Web Services (AWS), Salesforce, etc. (specify below)
- [ ] Other academic institution
- [ ] 3rd party vendor (business entity)
- [ ] Other (explain below)

* What online survey tool will you use: **(REQUIRED)**

- [x] Qualtrics (Recommended)
- [ ] RedCAP (Recommended)
- [ ] Survey Monkey (NOT recommended and may require UCSF ITS Security review)
- [ ] Other

12.19 * ADDITION OF RECORDS TO A REGISTRY: Will patient records reviewed under this approval be added to a research database, repository, or registry (either already existing or established under this protocol): (REQUIRED)

○ Yes ◉ No

12.20 * DATA SHARING: During the lifecycle of data collection, transmission, and storage, will identifiable information be shared with or be accessible to anyone outside of UCSF: (REQUIRED)

○ Yes ◉ No

13.0 Financial Considerations

13.1 * PAYMENT: Will subjects be paid for participation, reimbursed for time or expenses, or receive any other kind of compensation: (REQUIRED)

◉ Yes ○ No

13.2 PAYMENT METHODS: Subjects payment or compensation method (check all that apply):

Payments will be (check all that apply):

- [ ] Cash
- [ ] Check
- [ ] Gift card
- [ ] Debit card
- [ ] UCSF Research Subject Payment Card
- [ ] Reimbursement for parking and other expenses
- [x] Other:

Specify **other** payment/compensation method:

We will carry out a drawing, and participants will have an opportunity to win one of two 2-night hotel stays in Sonoma/Napa, valued at ~$1,000 each. In addition, all participants receive 1 year of access to the digital meditation program, valued at ~$150.

13.3 PAYMENT SCHEDULE: Describe the schedule and amounts of payments, including the total subjects can receive for completing the study:

- **If there are multiple visits over time, explain how payments will be prorated for partial completion**
- **If deviating from recommendations in Subject Payment Guidelines, include specific justification below**

All participant receive access to the digital meditation program for 1 year. Participants initially randomized to the digital meditation condition will begin their 1 year subscription at that time. Those in the waitlist condition will receive their 1 year subscription at the conclusion of the study (4-months after the baseline time point). Regarding the drawing, we will hold 2 drawings. The first will occur when half of the sample is recruited (or 1 year from first participant consent, which ever comes first) and the second drawing will occur when the final participant is recruited (or 3 years from first participant consent, which ever comes first).

13.4 COSTS TO SUBJECTS: Will subjects or their insurance be charged for any study activities:

○ Yes ◉ No

14.0 Other Approvals and Registrations

15.0 Qualifications of Key Study Personnel and External Personnel

NEW: January 2019 - External personnel who do not need access to UCSF networks and systems no longer need to get a UCSF ID. Add them below in the External Personnel table.

15.1

Instructions:

For UCSF Key Study Personnel (KSP)* added in Section 3, select the KSP from the drop down list and add a description of their study responsibilities, qualifications and training. In study responsibilities, identify every individual who will be involved in the consent process. Under qualifications, please include:

- Academic Title
- Institutional Affiliation (UCSF, SFGH, VAMC, etc.)
- Department
- Certifications

NOTE: This information is required and your application will be considered incomplete without it. If this study involves invasive or risky procedures, or procedures requiring special training or certification, please identify who will be conducting these procedures and provide details about their qualifications and training. Click the orange question mark for more information and examples.

Training Requirements:
The IRB requires that all Key Study Personnel complete Human Subjects Protection Training through **CITI** prior to approval of a new study, or a modification in which KSP are being added. More information on the CITI training requirement can be found on our **website**.

* **Definition of Key Study Personnel and CITI Training Requirements (Nov, 2015):** UCSF Key Study Personnel include the Principal Investigator, other investigators and research personnel who are directly involved in conducting research with study participants or who are directly involved in using study participants' identifiable private information during the course of the research. Key Personnel also include faculty mentors/advisors who provide direct oversight to Postdoctoral Fellows, Residents and Clinical Fellows serving as PI on the IRB application.

KSP Name	Description of Study Responsibilities - Briefly describe what will each person be doing on the study. If there are procedures requiring special expertise or certification, identify who will be carrying these out. Also identify who will be obtaining informed consent.	Qualifications, Licensure, and Training
Dr. Epel, Elissa Ph.D.	Co-Principal Investigator -- responsible for overseeing most aspects of the study, particularly the design, data management and analysis.	Dr. Epel has been managing clinical studies including intervention research such as this one for over 10 years.
Prather, Aric A, PhD	Principal Investigator -- responsible for overseeing all aspects of the study, including CHR management, implementation of data collection, analysis, and final reports.	Dr. Prather has a PhD in Clinical Psychology and is currently an Assistant Professor in the department of Psychiatry and co-director of the Center for Health and Community. He has been leading research studies on stress and sleep for over 5 years.
Crosswell, Alexandra D	Co-investigator-- will assist in study design and support in data analyses and more.	Dr. Crosswell has a PhD in Psychology and has lead and conducted similar research in meditation interventions.
Radin, Rachel M	Co-investigator-- will assist in analyzing measures of stress and health behaviors such as stress-eating.	Dr. Radin is a Post-doctoral scholar in the Center for Health and Community
Schilf, Samantha	Study Coordinator. Samantha will assist the study coordinator in	Samantha has worked in Drs. Epel and Prather's laboratory for five years. In this time she

	recruitment, participant experience, data management and more.	has gained knowledge and experience in clinical interventions.
Vaccaro, Julie M	Study Coordinator. Julie will assist the research team in carrying out research, from recruitment to data management and the informed consent process.	Julie has a B.S. in Psychology and volunteered as a Research Assistant. She works in Dr. Epel and Prather's lab.
Fisher, Sarah M	Study Coordinator. Sarah will assist the research team in carrying out research, from recruitment to data management and the informed consent process.	Sarah has a B.A. in Psychology and volunteered as a Research Assistant. She works in Dr. Epel and Prather's lab.
Fromer, Elena N	Research Assistant. Elena will assist the research team in carrying out research, from recruitment to data management and the informed consent process.	Elena has a B.S. in Psychology and volunteered as a Research Assistant. She works in Dr. Epel and Prather's lab.

15.2 Add any study personnel who are not associated with UCSF or one of our affiliated institutions (SF VAMC, Gladstone, Institute on Aging, etc.) and who will *NOT* need to log in to iRIS or access any UCSF networks or systems. Research support staff at affiliated institutions who will not need access to UCSF networks and systems can also be listed here.

Name	Institution	Telephone	E-mail	Role
No External Personnel has been added to this IRB Study				

Please describe the study responsibilities and qualifications of each external personnel listed above:

16.0 End of Study Application

End of Study Application Form

To continue working on the Study Application:
Click on the section you need to edit in the left-hand menu. Remember to save through the entire Study Application after making changes.

If you are done working on the Study Application:
Important: Before proceeding, please go back to Section 4.0 Initial Screening Questions and **Save and Continue** through the form to make sure all the relevant sections and questions have been included. If you've changed any answers since you started, the branching may have changed. Your application will be incomplete and it will have to be returned for corrections.

Once you are sure the form is complete, click **Save and Continue**. If this is a new study, you will automatically enter the **Initial Review Submission Packet Form**, where you can attach **consent forms** or other **study documents**. Review the **Initial Review Submission Checklist** for a list of required attachments.

Answer all questions and attach all required documents to speed up your approval.

The UCSF IRB welcomes feedback about the IRB Study Application Form. Please click the link to answer a **survey** about the application form.

Modification Form (Version 11.0)

1.0

Modification Form

March 2019 Version

This form features dynamic show/hide functionality. For the best experience, please avoid using the Chrome browser.

1.1 Principal Investigator:

Aric A Prather, PhD

1.2 Study Title:

Stress Free UCSF

1.3 Study Number:

17-23717

1.4 Lay Summary:

The aim of the present study is to test the effects of a digital meditation intervention in a sample of high stress UCSF employees. We propose to randomize 1000 UCSF employees to 8-weeks of either a digital mindfulness intervention (using the commerically available application Headspace) or a waitlist control condition. Measurement time points include baseline, week 4, week 8 (post intervention), and 4-month follow up period. Among participants who are randomized to the digital meditation intervention, they will also take part in a 1-year follow up. Our primary outcomes will be reductions in global levels of perceived stress and job strain. Secondary outcomes include improvements in mindfulness, symptoms of anxiety and depression, health behaviors, including sleep, physical activity, and eating behavior, and health care utilization.

Within this larger study, we will also conduct a smaller more focused substudy in which we will recruit_over weight and obese participants (BMI => 25). This study will include all of the measures obtained in the larger, main study but also expand to include physiological assessments, personal fitness and sleep metrics, and measures of daily mood data. The physiological assessments will take place at baseline and week 8, and the fitness, sleep, and mood data will be gathered for seven consecutive days at baseline and week 7. All other measurement time points are similar to those of the main study. For this substudy, we will recruit 150 additional UCSF employees, who will be randomly assigned to one of four conditions: digital meditation, a healthy eating intervention, digital meditation+healthy eating intervention, or a waitlist control condition. If participants are assigned to either of the Healthy Eating groups, in addition to the above-mentioned assessments, they will participate in one 30-minute counseling session centered around healthy eating behaviors such as decreasing sugar intake or the frequency of stress-related eating, and three 10-minute booster phone calls at week 1, week 4, and week 8.

1.5 * Modification level: (REQUIRED)

- ○ Administrative (fixing typos, changing study support staff, fixing consent form formatting, etc.)
- ◉ Minor
- ○ Major

Please see our online guidance for the difference between 'Major' and 'Minor' modifications

1.6 * **NEW PROTOCOL VERSION: Does this modification include a new version of the *Study Protocol* (e.g., the Sponsor's or Multicenter Protocol, *not* the IRB Study Application) OR a memo from the sponsor or cooperative group clarifying or changing procedures: (REQUIRED)**

○ Yes ◉ No

1.7 * **CHANGES TO CLINICAL ACTIVITIES: Does this modification add (or remove) any services or procedures (e.g. physical exams, surgeries, lab tests, imaging studies, or drugs) that could be billable to patients, their insurance, Sponsor, or any other entity (answer 'Yes' even if the study pays for all procedures): (REQUIRED) If you are not sure and need help deciding, contact the UCSF Office of Clinical Research at (415) 502-1521 or clinicaltrials@ucsf.edu.**

○ Yes ◉ No

1.8 * **Types of changes being made (check all that apply): (REQUIRED)**

- [ ] Adding newly identified risks
- [ ] Making changes to PI or personnel
- [ ] Adding a new funding source
- [ ] Adding sites
- [ ] Increasing enrollment numbers
- [ ] Making changes to imaging or procedures that involve radiation exposure (X-rays, CTs, DEXA, CT-guided biopsy, nuclear medicine including PET, MUGA, and bone scans)
- [ ] Modification to follow subject or subject's partner who became pregnant on study
- [x] Study changes, such as changes to recruitment, procedures, etc.
- [ ] Changes that don't affect the IRB application or documents
- [ ] Any other type of change (describe below)

If the other changes are related to information provided in the application and/or the consent form, please make these changes and attach the revised form / documents.

1.9 * **Are any of these changes being made as a result of an adverse event report (AER), protocol violation or incident report, or publication of a new Investigator's Brochure (IB) or other safety data: (REQUIRED)**

○ Yes ◉ No

1.12 * **Do any of these changes affect the Risk/Benefit ratio for participants, either favorably or unfavorably: (REQUIRED)**

○ Yes ◉ No

1.13 **List any minor and/or administrative changes and provide justifications where appropriate:**
Important: Click the 'Save' button at the top of this form after you've typed your answer to prevent loss of work.

1. For participants randomized to the "Healthy Eating" or "Headspace + Healthy Eating" conditions, the counseling session will last anywhere from 30 to 60 minutes. The previous version specified the session would last 30 minutes.

2. For participants randomized to the "Healthy Eating" or "Headspace + Healthy Eating" conditions, they will be required to access the online audio exercises at least once per week over the course of their 8-week participation in the study. The previous version specified that these exercises were optional.

1.14 REVISED STUDY APPLICATION:

* Does this modification require changes to the Study Application: **(REQUIRED)**

◉ Yes ○ No

Click the bar below to attach a revised application.

Important: Click the 'Save' button at the top of this form to save your answers above before clicking the bar to attach the application.

Edit/ View	Version	Title
	1.12	Study Application (Version 1.12) - Attached

Please use the compare forms function in iRIS to ensure that the only differences between this version and the last approved version directly correlate to the site-related changes described in this form. Your submission will be returned if it includes any additional changes not listed above.

1.15 NEW AND/OR REVISED DOCUMENTS AND CONSENT FORMS:

* Do you need to attach any revised consent forms: **(REQUIRED)**

○ Yes ◉ No

Version	Sponsor Version	Title	Category	Language	Expiration Date	Consent Outcome	View Document
No Consent(s) have been attached to this form.							

* Do you need to attach any new or revised Study Documents: **(REQUIRED)**

○ Yes ◉ No

Version	Sponsor Version	Title	Category	Expiration Date	Document Outcome	View Document
No Document(s) have been attached to this form.						

1.16 Check here if this is modification includes adding the use of CTSI Clinical Research Services or

Centers for the first time:

○ Adding CRS

1.18 * Are there any changes in any financial interests related to this study or in any conflicts of interest of the PI or any other investigator?

○ Yes ◉ No

If **Yes**, the Conflict of Interest Advisory Committee (COIAC) office may contact you for additional information.

1.19 Outstanding Stipulations:

No Stipulation is outstanding.

1.20 THIS IS THE END OF THE FORM Click 'Save and Continue to the Next Section' to get to the 'Signoff and Submit' screen.
