# Supplementary material for: Impact of digital meditation on work stress and health outcomes among adults with overweight: A randomized controlled trial
Source: PLoS One. 2023 Mar 1;18(3):e0280808. doi: 10.1371/journal.pone.0280808 (PMC9977041; doi:10.1371/journal.pone.0280808)
Supplement: Attachment — Submitted filename: Response to Reviewers.pdf [file pone.0280808.s006.pdf]

UNIVERSITY OF CALIFORNIA, SAN FRANCISCO

BERKELEY • DAVIS • IRVINE • LOS ANGELES • RIVERSIDE • SAN DIEGO • SAN FRANCISCO • SANTA BARBARA • SANTA CRUZ

Rachel M. Radin, PhD
Assistant Professor
Department of Psychiatry and Behavioral Sciences
University of California, San Francisco
Rachel.Radin@ucsf.edu

Dear Dr. Tischer,

We are pleased to submit a revised version of our research article, entitled *Impact of digital meditation on work stress and health outcomes among adults with overweight: A randomized controlled trial* for consideration of publication in PLOS ONE. We are grateful for the reviewers' thoughtful feedback and have included our detailed responses below. We have also highlighted changes throughout our manuscript using tracked changes.

Response to Editor and Reviewer Comments:

Editor's comments:

- Provision of CONSORT checklist- **this is now included**
- Include marked up copy of manuscript with tracked changes as well as unmarked version without tracked changes- **these are now included**

Reviewer #1:

- *This review pertains only to 'statistical aspects' of the study and so 'clinical aspects' [like medical importance, relevance of the study, 'clinical significance and implication(s)' of the whole study, etc.] are to be evaluated [should be assessed] separately/independently. Further please note that any 'statistical review' is generally done under the assumption that (such) study specific methodological [as well as execution] issues are perfectly taken care of by the investigator(s). This review is not an exception to that and so does not cover clinical aspects (however, seldom comments are made only if those issues are intimately / scientifically related & intermingle with 'statistical aspects' of the study). Agreed that 'statistical methods' are used as just tools here, however, they are vital part of methodology [and so should be given due importance].*
    - **RESPONSE: Noted. We appreciate this reviewer's keen insights with regard to statistical aspects of this study. We feel this reviewer's feedback has greatly enhanced the quality and rigor of the manuscript.**
- It is definitely a good study and is planned as well as executed nicely.
    - **RESPONSE: We thank this reviewer for your positive feedback regarding the manuscript's methodology.**
- Why there were two control groups [active control ('HE,' n=41), or waitlist control ('WL,' n=42)] in the study?' [You may know that 'Permuted Block Randomization' ensures same group sizes (not]. Role/importance/necessity of 'waitlist controls' in any Psychology study is well-known. However, why two control groups?
    - **RESPONSE: We thank this reviewer for this important question. We felt that the addition of an active control group ('healthy eating') which did not include a general mindfulness component, but which was matched for time and attention and other non-specific intervention effects, would allow us to test whether meditation could "out-perform" both a waitlist control (with no treatment or attention components whatsoever) and an active control (that featured some clinical attention and some general health guidelines around eating, generally thought to be a non-specific treatment component for improving eating behavior). The issue of adding an active control group to clinical trials has been receiving more and more attention in recent**

years, particularly in psychological intervention research, and is generally considered advantageous to a purely waitlist control condition (Kinser & Robins, 2013). **We have added this clarification to lines 102-103:**
    - **We aimed to test whether digital meditation could out-perform an active control condition that was matched for time and attention and other non-specific intervention effects (Kinser & Robins, 2013).**

- Tool used to measure 'perceived stress' should have been mentioned in the abstract itself [as it is a Primary Outcome Measure]. In the 'Abstract-Results' section you said "Those with greater adherence to MED or MED+HE had greater reductions in stress [, cravings, and adiposity (ps<.05)]" but not mentioned tool or table (where this change is displayed and tested). Much later in line 156, you mentioned that the Perceived Stress Scale (PSS) was used. Is that alright?
  - **RESPONSE: We thank this reviewer for noting this oversight. We have added more specificity about primary and secondary outcomes measures to the abstract, on lines 30-31:**
    - **Participants (*n*=145; M(SD) BMI: 30.8 (5.4) kg/m^2) completed baseline and 8-week measures of stress (Perceived Stress Scale), cravings (Food Acceptance and Awareness Questionnaire) and adiposity (sagittal diameter and BMI).**
- The Food Acceptance and Awareness Questionnaire (FAAQ) was used to measure acceptance of urges and cravings to eat. Since the FAAQ is (made up of 10 items, each) rated on a 6-point Likert scale (1=very seldom true to 6=always true) and might have included 'not true' (negative) response also, which needs reverse scoring (very often). Also in this context, please note that the following (which is pasted from one standard textbook on 'Research Methodology' and I am sure that the authors already know these things, however, it is very essential to keep the limitations in mind while interpreting results [note that I am not asking you to change the study design]: Whenever response options ranged from 1=strongly disagree to 4=strongly agree (or ranging from 1 (strongly disagree) to 6 (strongly agree) or from 1=very bad to 3=neither good nor bad to 5=very good), while using a 'Likert' scale responses, recoding [like strongly disagree=-2, disagree=-1, neutral=0, agree=1, strongly agree=2] may yield correct and meaningful 'arithmetic mean' which is useful not only for comparison but has absolute meaning, in my opinion. Application of any statistical test(s) assume that meaning of entity used (mean, SD, etc) has a particular meaning. Though 'α' [alpha] or most other measures of reliability/correlation will remain same, however, use of non- parametric methods should/may be preferred while dealing with data yielded by any questionnaire/score.
  - **RESPONSE: We thank this reviewer for noting the limitation of the way in which the FAAQ is scored using a 6-point Likert scale, without the option for negative or not true responses. We have kept the scoring to be consistent with conventional scoring approaches for this measure (Juarascio, Forman, Timko, Butryn, & Goodwin, 2011), to ensure ease of comparison of FAAQ scores across manuscripts. However, we acknowledge this scoring limitation in our discussion section, on lines 467-470:**
    - **Further, our measures of dysregulated eating may not fully reflect non-homeostatic eating behavior (vs. a semi-structured interview measure of eating pathology), and the scoring metrics for the FAAQ (a 6-point Likert scale ranging from 1 to 6) without the option for including negative (e.g., -1, -2) response may not yield particularly meaningful arithmetic means.**
- Further note that though the measures/tools used are appropriate, most of them yield data that are in [at the most] 'ordinal' level of measurement [and not in ratio level of measurement for sure (as the score two times higher does not indicate presence of that parameter/phenomenon as double (for example, a Visual Analogue Scales VAS score or say 'depression/stress' score)]. Then application of suitable non-parametric test(s) is/are indicated/advisable [even if distribution may be 'Gaussian' (i.e., normal)]. Agreed that there is/are no non-parametric test(s)/technique(s) available to be used as alternative in all situation(s) [suitable / most desired/applicable], but should be used whenever/wherever they are available.
  - **RESPONSE: We thank this reviewer for noting the limitation of survey measures that yield data in the ordinal level of measurement (e.g., PSS, FAAQ). Per this reviewer's suggestion, we have run non-parametric tests (Kruskal-Wallis test, instead of ANOVA) for outcome measures using ordinal level measurement (PSS and FAAQ) where possible, and yielded nearly identical findings as with our parametric tests. Due to space limitations we have not included these findings in the manuscript, but we acknowledge the following on lines 322-323, and lines 342-343:**

        - **Findings were identical when using non-parametric tests (Kruskal-Wallis), given the ordinal nature of the PSS scoring.**
        - **Findings were identical when using non-parametric tests (Kruskal-Wallis), given the ordinal nature of the FAAQ scoring.**
- As you know well that while reporting [findings from] 'Clinical Trial' one should follow CONSORT guidelines. Even important items (like How sample size was determined (Item 7a), Random Sequence generation (Item 8a), Allocation concealment (Item 9), Blinding (Item 11a)) of/in CONSORT checklist are not found [since your article type is 'Clinical Trial', you are supposed to cover these items in the report]. How you arrived at this sample size [with complete estimation procedure] must be described in details as ultimately you had to say (lines 371-2 that 'our study was likely limited by a sample size that may have been too small to detect modest interaction effects'). Fig 1. CONSORT Flow Diagram is alright but covers only about flow of cases/numbers.
    - **RESPONSE: We thank this reviewer for noting this oversight. Most of these details required by CONSORT guidelines are contained within our Supplemental CONSORT Study protocol, which has now been submitted to the editor. We have also added most of these details throughout the manuscript. For instance, on lines 133-136 and on lines 142-148, respectively, we have added the following:**
        - **We aimed to enroll up to 150 participants. Our prior study** (Bostock, Crosswell, Prather, & Steptoe, 2019) **detected effects in a sample of <250 participants. We therefore expected that our sample size of 150 would be well-powered to detect improvements in our self-report measures in response to our treatment intervention.**
        - **Study personnel then randomly assigned participants to one of four possible conditions, using factorial assignment, on Qualtrics…. Study personnel were not able to access the file containing the sequence of assignments or to see the next condition in the sequence until the moment they randomized the participant.**
- There are only two tables in the manuscript – one on baseline demographics and the other on baseline heath characteristics – remaining vital information [mainly comparison statistics] are put/presented in either text or figures. But remember that (in my opinion) figures are complementary and not alternatives of/to tables. One good thing is that there is no statistical comparison of baseline characteristics [read the following]:
  To provide a description of baseline characteristics is entirely reasonable (since it is clearly important in assessing to whom the results of the trial can be applied), however, statistical comparison of baseline characteristics is not desirable at all [because even if P-value turns out to be significant (while comparing baseline characteristics despite random allocation), it is, by definition, a false positive] as you then are supposed to be testing 'randomization' then, which in any single trial may not balance all baseline characteristics [particularly when sample sizes are small] because 'randomization' is a sort of 'insurance' and not a guarantee scheme.
    - **RESPONSE: We thank this reviewer for this comment and completely agree that baseline characteristics should not include any sort of statistical comparison. We also agree with this reviewer that figures are considered complementary, and have thus included all relevant and necessary statistics pertaining to these figures within the text of the manuscript.**
- Is not it essential to adjust P-value(s) even if 'series of Analysis of Covariance (ANCOVA)' are used/applied as it a sort of multiple testing (multiple comparisons) problem/issue?
    - **RESPONSE: We thank this reviewer for this keen observation regarding multiple comparisons using ANCOVA. Notably, all of our results remained similar when adjusting for multiple comparisons (using a Bonferroni adjusted alpha level of .03 (.05/2) for each ANCOVA model).**
- Except these few points, this manuscript is alright and I have no hesitation to recommend acceptance after minor revision.
    - **RESPONSE: We thank this reviewer for your positive feedback regarding the overall quality of the manuscript, and appreciate the keen attention to statistical concerns throughout.**

<u>Reviewer #2</u>:

- Title: The coma (,) in the randomized controlled trial shall be excluded.
    - **RESPONSE: We thank this reviewer for noting this oversight and have removed the comma from the title (Line 4).**

- *Materials and Methods:*
- Under "Interventions", please specify the frequency (e.g.. on a daily basis or at least N number of days per week of the 8-weeks intervention.
    - **RESPONSE: We have added frequency of contact/involvement for each intervention category. Lines 155-186 now say:**
        - **Meditation group ('MED')…Participants were expected to meditate 5 days per week over the course of 8 weeks**
        - **Healthy eating group ('HE')… Participants had a total of approximately 1.5-2 hours of contact with a counselor, and were expected to engage with the online resources 1 day per week over the course of 8 weeks.**
        - **Meditation + Healthy eating group ('MED+HE')… Participants were expected to meditate 5 days per week over the course of 8 weeks and they had a total of approximately 1.5-2 hours of contact with a counselor, and were expected to engage with the online resources 1 day per week over the course of 8 weeks.**
        - **Waitlist control condition ('WL')… Participants had no contact with a study counselor over the course of the 8 week intervention period.**
- Also, please include how the researchers have verified whether the participants had used the app in the given period.
    - **RESPONSE: The research team had access to user data through Headspace, and were able to calculate total number of minutes spent meditating. This information has been added to lines 238-239:**
        - **The research team had access to individual user data via Headspace, in order to make these calculations.**
- For HE Group, when was the counseling conducted? Was it at the beginning of the intervention?
    - **RESPONSE: The counseling session was conducted at the very beginning of the intervention, within week one. This information has been added to line 158.**
- Please elaborate on the "digitally-based mindful eating program." Include name of the app, duration of the mindful eating practice and how the usage per user was assured.
    - **RESPONSE: The digitally-based mindful eating program was created specifically for this study by the research team, and was primarily a secured website that included information on mindful eating, and audio tools for mindful eating practice (~3-5 minute practices). These details have now been added to lines 164-166.**
- Did you check if the waitlist control group had already access to Headspace or other mindfulness apps like Calm? Have you also considered previous experience of (all) the participants with regard to mindfulness or other meditation practices?
    - **RESPONSE: We did not provide Headspace access codes to WL participants until after they completed a 2-month follow-up questionnaire. However, we had no way of objectively verifying whether participants already had subscriptions to other mindfulness apps. We excluded individuals who indicated they were experienced meditators (defined as 3 times per week for 10 minutes or more), and at baseline, participants reported meditating less than once a week; in fact, the majority (79%) indicated that they had never meditated, and only 4% indicated that they meditated 1-2 times per week prior to treatment randomization (Table 2). Given that so few individuals indicated prior experience with meditation, we did not examine this baseline characteristic as a treatment covariate. We have noted the limitation of being unable to objectively verify access to meditation programs for those in our control conditions to lines 465-467:**
        - **We were unable to truly ascertain whether participants in either control condition were accessing mindfulness programs or apps during the 8 week intervention period.**
- Under "Measures", please include the reliability and validity of the instruments used.
    - **RESPONSE: We appreciate this feedback, and we have now included relevant psychometric properties of each of the instruments used (PSS, FAAQ, and QEWP-5) throughout the measures section (Lines 193-231).**
- *Discussion and Conclusion:*
- Please separate Conclusion as a distinct section.

- **RESPONSE: We have now created a Conclusion section that is distinct from the Discussion section (Line 473), and which highlights the clinical implications of the study.**

Sincerely yours,

Rachel M. Radin

Rachel M. Radin, PhD

References Cited

Bostock, S., Crosswell, A. D., Prather, A. A., & Steptoe, A. (2019). Mindfulness on-the-go: Effects of a mindfulness meditation app on work stress and well-being. *Journal of Occupational Health Psychology, 24*(1), 127.

Juarascio, A., Forman, E., Timko, C. A., Butryn, M., & Goodwin, C. (2011). The development and validation of the food craving acceptance and action questionnaire (FAAQ). *Eat Behav, 12*(3), 182-187. doi:10.1016/j.eatbeh.2011.04.008

Kinser, P. A., & Robins, J. L. (2013). Control group design: enhancing rigor in research of mind-body therapies for depression. *Evid Based Complement Alternat Med, 2013*, 140467. doi:10.1155/2013/140467
