# Supplementary material for: Impact of digital meditation on work stress and health outcomes among adults with overweight: A randomized controlled trial
Source: PLoS One. 2023 Mar 1;18(3):e0280808. doi: 10.1371/journal.pone.0280808 (PMC9977041; doi:10.1371/journal.pone.0280808)
Supplement: Attachment — Submitted filename: renamed_6f0b7.docx [file pone.0280808.s004.docx]

**COMMENTS – Manuscript PONE-D-21-38719**

Title: “Impact of digital meditation on work stress and health outcomes among adults with overweight: A randomized, controlled trial”

**Important note: This review pertains only to ‘statistical aspects’ of the study and so** **‘clinical aspects’ [like medical importance,** **relevance of the study,** **‘clinical significance and implication(s)’ of the whole study, etc.] are to be evaluated [should be assessed] separately/independently. Further please note that any ‘statistical review’ is generally done under the assumption that (such) study specific methodological [as well as execution] issues are perfectly taken care of by the investigator(s). This review is not an exception to that and so does not cover clinical aspects {however, seldom comments are made only if those issues are intimately / scientifically related & intermingle with ‘statistical aspects’ of the study}. Agreed that ‘statistical methods’ are used as just tools here, however, they are vital part of methodology [and so should be given due importance].**

**COMMENTS:** It is definitely a good study and is planned as well as executed nicely. However, I have few doubts [these respected actions may be correct but need explanations/clarifications/justifications. Please take them as suggestions]. The first is: ‘Why there were two control groups [active control (‘HE,’ *n*=41), or waitlist control (‘WL,’ n=42)] in the study?’ [You may know that ‘Permuted Block Randomization’ ensures same group sizes (not]. Role/importance/necessity of ‘waitlist controls’ in any Psychology study is well-known. However, why two control groups?

Next is: Tool used to measure ‘perceived stress’ should have been mentioned in the abstract itself [as it is a Primary Outcome Measure]. In the ‘Abstract-Results’ section you said “Those with greater adherence to MED or MED+HE had greater reductions in stress [, cravings, and adiposity (*ps*<.05)]” but not mentioned tool or table (where this change is displayed and tested). Much later in line 156, you mentioned that the Perceived Stress Scale (PSS) was used. Is that alright?

The Food Acceptance and Awareness Questionnaire (FAAQ) was used to measure acceptance of urges and cravings to eat. Since the FAAQ is {made up of 10 items, each} rated on a 6-point Likert scale (1=very seldom true to 6=always true) and might have included ‘not true’ (negative) response also, which needs reverse scoring (very often). Also in this context, please note that the following {which is pasted from one standard textbook on ‘Research Methodology’ and I am sure that the authors already know these things, however, it is very essential to keep the limitations in mind while interpreting results [note that I am not asking you to change the study design]}:

Whenever response options ranged from 1=strongly disagree to 4=strongly agree (or ranging from 1 (strongly disagree) to 6 (strongly agree) or from 1=very bad to 3=neither good nor bad to 5=very good), while using a ‘Likert’ scale responses, recoding [like strongly disagree=-2, disagree=-1, neutral=0, agree=1, strongly agree=2] may yield correct and meaningful ‘arithmetic mean’ which is useful not only for comparison but has absolute meaning, in my opinion. Application of any statistical test(s) assume that meaning of entity used (mean, SD, etc) has a particular meaning. Though ‘α’ [alpha] or most other measures of reliability/correlation will remain same, however, use of non-parametric methods should/may be preferred while dealing with data yielded by any questionnaire/score.

Further note that though the measures/tools used are appropriate, most of them yield data that are in [at the most] ‘ordinal’ level of measurement [and not in ratio level of measurement for sure {as the score two times higher does not indicate presence of that parameter/phenomenon as double (for example, a Visual Analogue Scales VAS score or say ‘depression/stress’ score)}]. Then application of suitable non-parametric test(s) is/are indicated/advisable [even if distribution may be ‘Gaussian’ (i.e., normal)]. Agreed that there is/are no non-parametric test(s)/technique(s) available to be used as alternative in all situation(s) [suitable / most desired/applicable], but should be used whenever/wherever they are available.

As you know well that while reporting [findings from] ‘Clinical Trial’ one should follow CONSORT guidelines. Even important items {like How sample size was determined (Item 7a), Random Sequence generation (Item 8a), Allocation concealment (Item 9), Blinding (Item 11a)} of/in CONSORT checklist are not found [since your article type is ‘Clinical Trial’, you are supposed to cover these items in the report]. How you arrived at this sample size [with complete estimation procedure] must be described in details as ultimately you had to say (lines 371-2 that ‘our study was likely limited by a sample size that may have been too small to detect modest interaction effects’}. Fig 1. CONSORT Flow Diagram is alright but covers only about flow of cases/numbers.

There are only two tables in the manuscript – one on baseline demographics and the other on baseline heath characteristics – remaining vital information [mainly comparison statistics] are put/presented in either text or figures. But remember that (in my opinion) figures are complementary and not alternatives of/to tables. One good thing is that there is no statistical comparison of baseline characteristics [read the following]:

To provide a description of baseline characteristics is entirely reasonable (since it is clearly important in assessing to whom the results of the trial can be applied), however, statistical comparison of baseline characteristics is not desirable at all [because even if P-value turns out to be significant (while comparing baseline characteristics despite random allocation), it is, by definition, a false positive] as you then are supposed to be testing ‘randomization’ then, which in any single trial may not balance all baseline characteristics [particularly when sample sizes are small] because ‘randomization’ is a sort of ‘insurance’ and not a guarantee scheme.

Is not it essential to adjust P-value(s) even if ‘series of Analysis of Covariance (ANCOVA)’ are used/applied as it a sort of multiple testing (multiple comparisons) problem/issue?

Except these few points, this manuscript is alright and I have no hesitation to recommend acceptance after minor revision.
