# Supplementary material for: Impact of digital meditation on work stress and health outcomes among adults with overweight: A randomized controlled trial
Source: PLoS One. 2023 Mar 1;18(3):e0280808. doi: 10.1371/journal.pone.0280808 (PMC9977041; doi:10.1371/journal.pone.0280808)
Supplement: Attachment — Submitted filename: COMMENTS.docx [file pone.0280808.s005.docx]

**Title**: The coma (,) in the randomized controlled trial shall be excluded.

**Materials and Methods**:

Under “Interventions”, please specify the frequency (eg. on a daily basis or at least N number of days per week of the 8-weeks intervention. Also, please include how the researchers have verified whether the participants had used the app in the given period.

For HE Group, when was the counseling conducted? Was it at the beginning of the intervention?

Please elaborate on the “digitally-based mindful eating program”. Include name of the app, duration of the mindful eating practice and how the usage per user was assured.

Did you check if the waitlist control group had already access to Headspace or other mindfulness apps like Calm? Have you also considered previous experience of (all) the participants with regard to mindfulness or other meditation practices?

Under “Measures”, please include the reliability and validity of the instruments used.

**Discussion and** **Conclusion**: Please separate Conclusion as a distinct section. Highlight the implications of the study at the end of Discussion, and also include delimitations.
