# Supplementary material for: Impact of digital meditation on work stress and health outcomes among adults with overweight: A randomized controlled trial
Source: PLoS One. 2023 Mar 1;18(3):e0280808. doi: 10.1371/journal.pone.0280808 (PMC9977041; doi:10.1371/journal.pone.0280808)
Supplement: Attachment — Submitted filename: Response to Reviewers_2022-11-17.docx [file pone.0280808.s007.docx]

| UNIVERSITY OF CALIFORNIA, SAN FRANCISCO    BERKELEY DAVIS IRVINE LOS ANGELES RIVERSIDE SAN DIEGO SAN FRANCISCO SANTA BARBARA SANTA CRUZ    Rachel M. Radin, PhD  Assistant Professor  Department of Psychiatry and Behavioral Sciences  University of California, San Francisco  [Rachel.Radin@ucsf.edu](mailto:Rachel.Radin@ucsf.edu) |  |
| --- | --- |

Dear Dr. McFadden,

We are pleased to submit a revised version of our research article, entitled *Impact of digital meditation on work stress and health outcomes among adults with overweight: A randomized controlled trial* for consideration of publication in PLOS ONE. We are pleased to learn that the previous peer reviewers were satisfied with our edits and responsiveness to earlier feedback. We are grateful for your thoughtful additional feedback and have included our detailed responses below. We have also highlighted changes throughout our manuscript using tracked changes.

**Response to Editor Comments:

Editor’s queries:**

- The manuscript mentions a "digitally-based mindful eating program" which was created for this study and provided to participants via a secured website. However, it is not clear what this program entailed, how it was designed and if it is intended to be provided as a commercial product. Please respond to the editor's queries regarding this issue. Please provide further information on the “digitally-based mindful eating program.” Please include in the manuscript a brief outline of the material in the digitally-based mindful eating program and how it was designed.
  - **RESPONSE: We developed a brief mindful eating program for this study, geared towards helping participants develop goals to improve eating behavior (e.g., to reduce compulsive eating and increase mindful eating). This program was in the early, pilot stages (NCT03945214, funded through the first author’s K23: 1K23AT011048) to assess feasibility and acceptability. Given its early stages of development, the program is not intended to be provided as a commercial product. It is in need of further refinement and tailoring for specific populations who struggle with binge eating before it can be offered as a commercial product. The program included a combination of both an in-person motivational-interviewing-based counseling session, text message support, and access to a website that provided audio exercises and tools to practice eating mindfully, including tools to ride out urges to overeat. The audio exercises were scripted and recorded by the study’s first author and adapted from a number of mindful eating resources including the MB-EAT curriculum.**

**We developed the brief mindful eating program for this study, geared towards helping participants develop goals to reduce compulsive eating and increase mindful eating. This program was in the pilot stages to assess feasibility and acceptability. The program is adapted from several sources, including motivational interviewing for binge eating,^1^ weight management,^2,3^ and sugar-sweetened beverage intake (from our recently completed trial),^4^ and mindfulness-based eating awareness training.^5^ The program is comprised of an initial one-on-one counseling session, three booster calls during the 8-week intervention period, engagement with an online mindful eating program with instruction on mindful eating practices, and text message reminders of support three times a week. During the session, trained health counselors follow a semi-structured protocol of: (1) engaging participants in a conversation about the concerns they have about their eating (2) identifying vulnerabilities for challenging eating patterns, (3) asking participants to consider why/how making changes to eating would be important for them, (4) identifying perceived barriers to changing eating habits, (5) providing psychoeducation on sugar intake, stress eating, and their links with health, (6) collaborating with participants on specific, achievable eating-related goals, (7) identifying motivational factors, (8) engaging participants to rate level of confidence and self-efficacy in making changes, and (9) guiding participants through a brief mindful eating exercise using a bite-sized snack of their choice. Participants were then given access to a password-secured website which contained up to six different brief audio exercises using mindful eating and urge-surfing strategies. They were instructed to access these audios during high vulnerability times for compulsive eating. For example, for those who identify cravings as a potent trigger for problematic consumption, participants could access a brief urge-surfing exercise to learn how to ‘ride out’ a craving.**

**The following details about the digital component of the program have now been added to the manuscript (lines 157-163):**

- **This password-secured website contained up to six different brief audio exercises using mindful eating and urge-surfing strategies. The audio exercises were scripted and recorded by the study’s first author and adapted from a number of mindful eating resources including the MB-EAT curriculum. We instructed participants to access these audios during high vulnerability times for compulsive eating. For example, for those who identify cravings as a potent trigger for problematic consumption, participants could access a brief urge-surfing exercise to learn how to ‘ride out’ a craving.**
- Are there any previous publications describing the program or research that underpins it? If so please cite them in the manuscript.
  - **RESPONSE**: **The program is adapted from several sources, including motivational interviewing for binge eating,^1^ weight management,^2,3^ and sugar-sweetened beverage intake (from our recently completed trial),^4^ and mindfulness-based eating awareness training.^5^ However, the current manuscript is the first to describe the program in its current form. The following details have now been added to the manuscript (lines 165-167):**
    - **The program is adapted from several sources, including motivational interviewing for binge eating,^1^ weight management,^2,3^ and sugar-sweetened beverage intake (from our recently completed trial),^4^ and mindfulness-based eating awareness training.^5^**
- Is there any public-facing information about the program accessible to readers? If so, please add links to relevant webpages as citation(s). Guidelines for formatting references to online sources are here: hfps://journals.plos.org/plosone/s/submission-guidelines#loc-references
  - **RESPONSE: This program was in the early, pilot stages (NCT03945214, funded through the first author’s K23: 1K23AT011048) to assess feasibility and acceptability. Given its early stages of development, the program is not yet accessible to readers in public-facing formats such as public webpages.**
- Is the program associated with a commercial provider and/or patent or is it intended to be provided commercially in the future? If so, are any of the authors associated with the commercial provider or patent? Please review PLOS's Competing Interest policy and ensure all potential competing interests are declared (hfps://journals.plos.org/plosone/s/compeUng-interests)
  - **RESPONSE: As described previously, this program was in the early, pilot stages (NCT03945214, funded through the first author’s K23: 1K23AT011048) to assess feasibility and acceptability. Given its early stages of development, the program is not intended to be provided as a commercial product. It is in need of further refinement and tailoring for specific populations who struggle with binge eating before it can be offered as a commercial product.**

Sincerely yours,

Rachel M. Radin, PhD
